# Supplementary figures and images for: Quiescent neural stem cells transiently become neuron-like to coordinate long-range reactivation
Source: EMBO J. 2026 Apr 24;45(11):3788–807. doi: 10.1038/s44318-026-00775-3 (PMC13226747; doi:10.1038/s44318-026-00775-3)

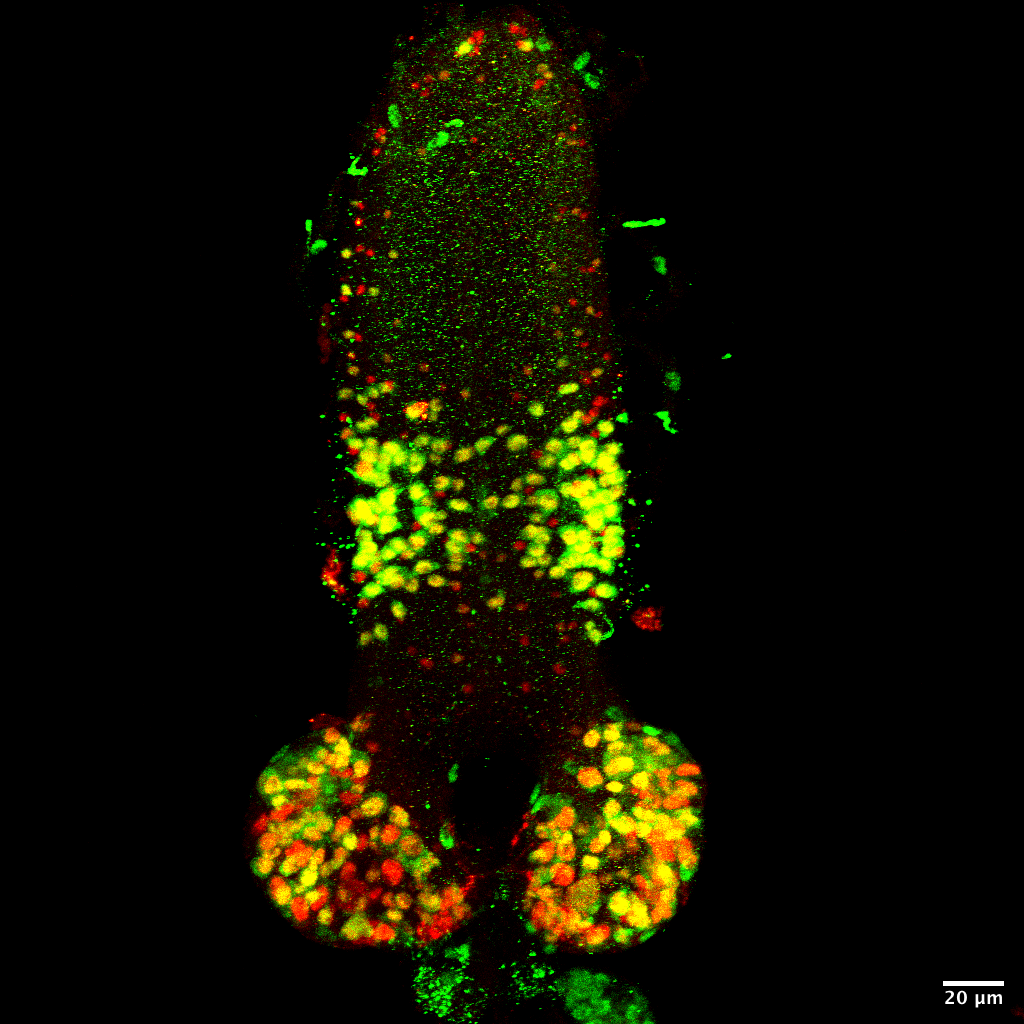

Supplement: Supplementary file 2 — Source data Fig. 1 [file 44318_2026_775_MOESM2_ESM.zip › Figure 1/1C/16hrs_CycADpn.tif]

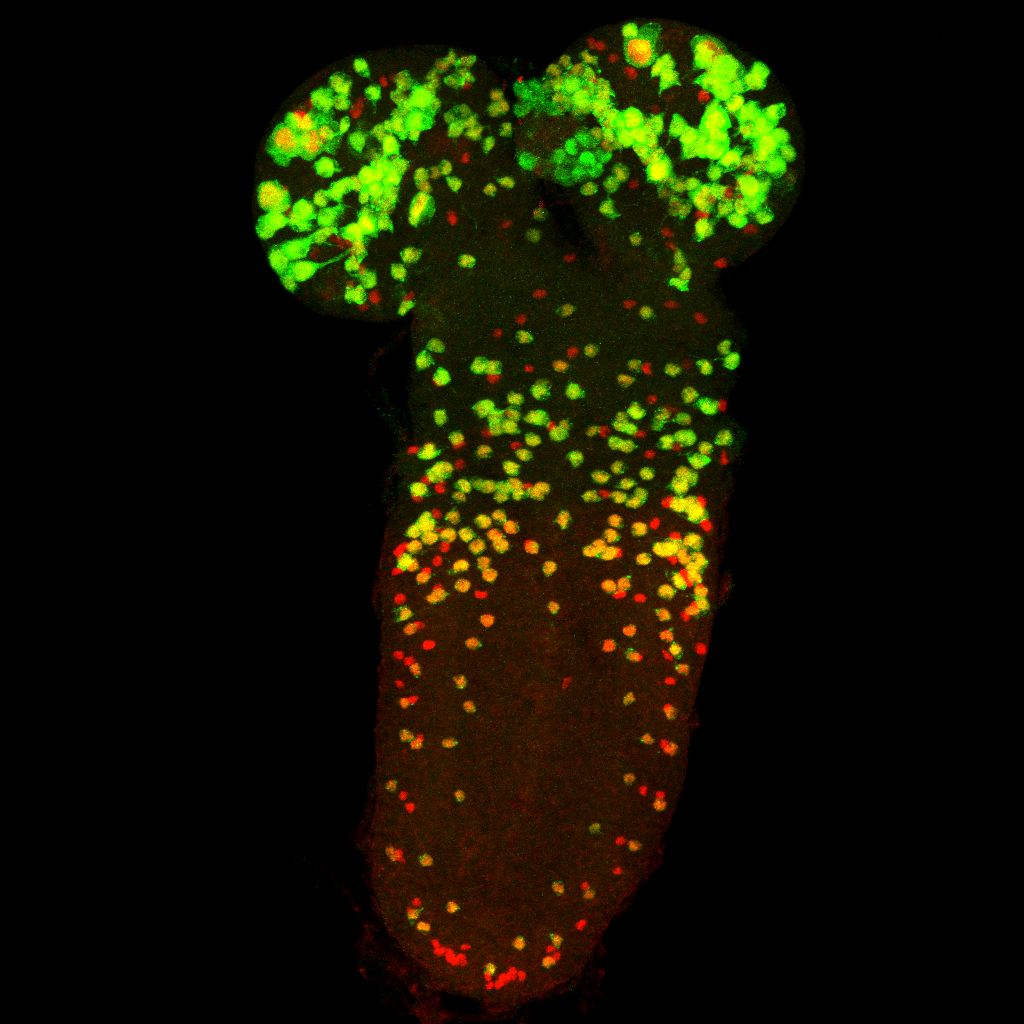

Supplement: Supplementary file 2 — Source data Fig. 1 [file 44318_2026_775_MOESM2_ESM.zip › Figure 1/1C/8hrs_CycADpn.tif]

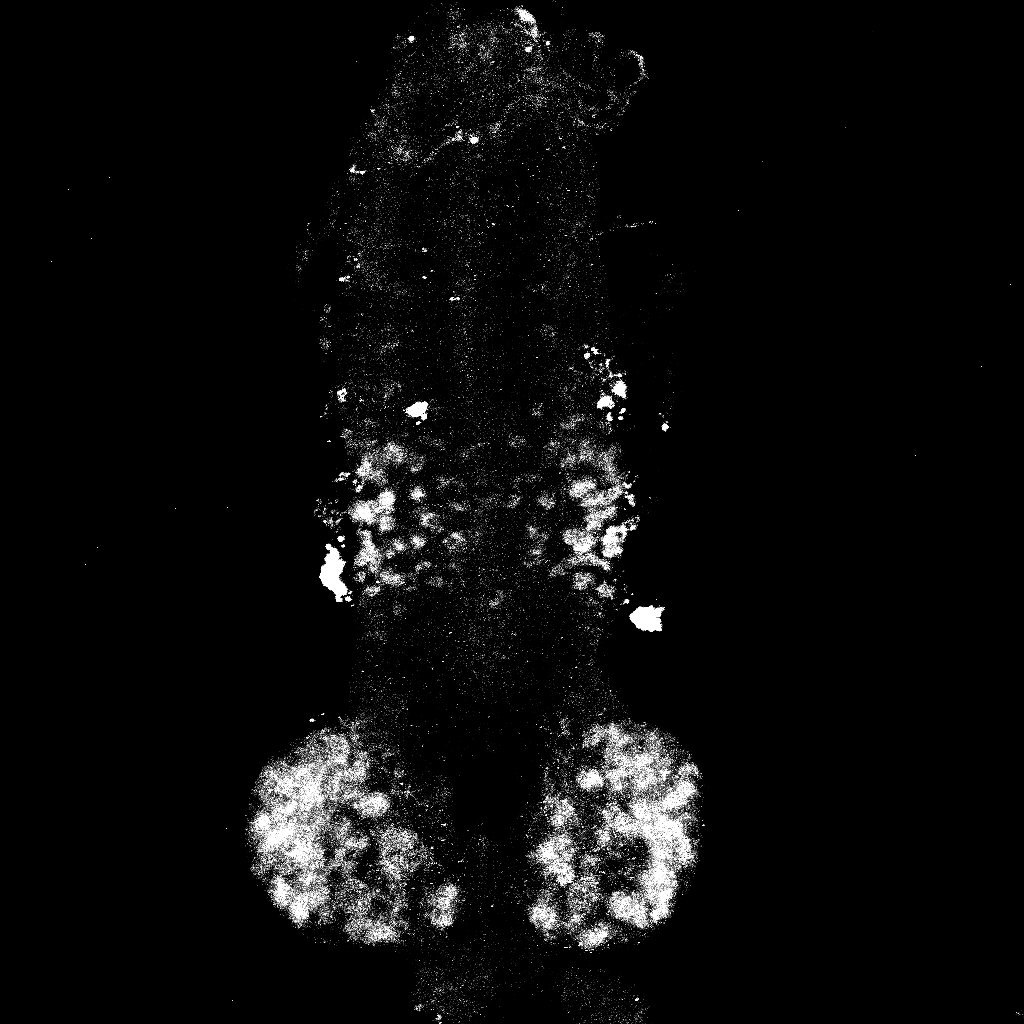

Supplement: Supplementary file 2 — Source data Fig. 1 [file 44318_2026_775_MOESM2_ESM.zip › Figure 1/1C/16hrs_Wor.tif]

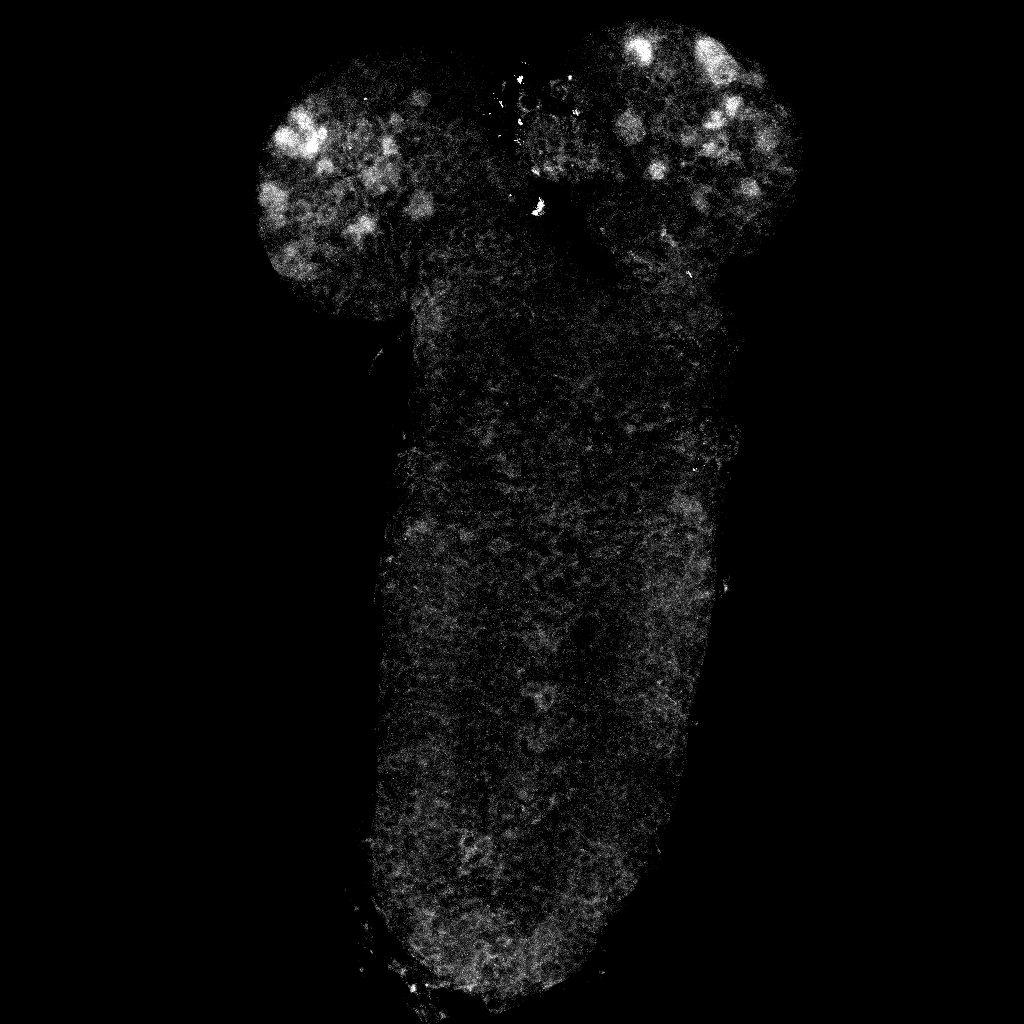

Supplement: Supplementary file 2 — Source data Fig. 1 [file 44318_2026_775_MOESM2_ESM.zip › Figure 1/1C/8hrs_Wor.tif]

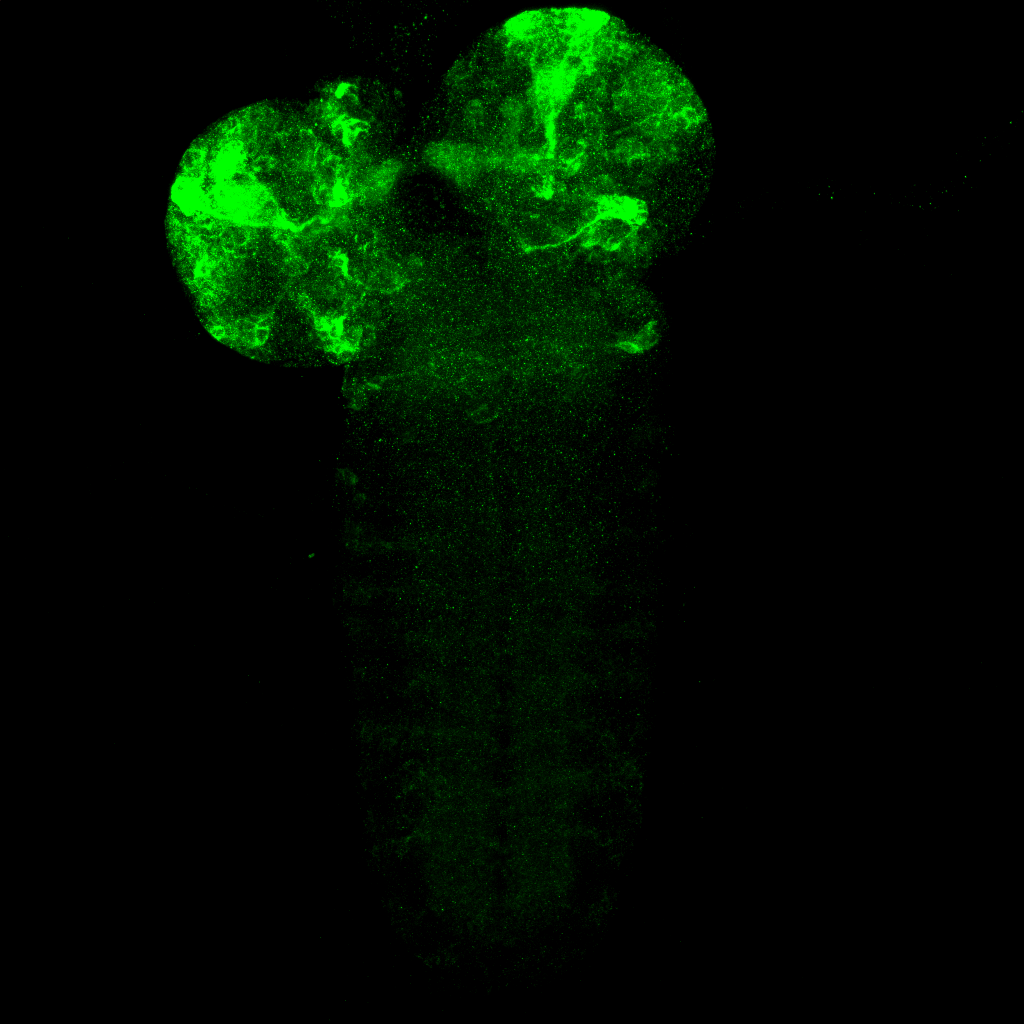

Supplement: Supplementary file 3 — Source data Fig. 2 [file 44318_2026_775_MOESM3_ESM.zip › Figure 2/2A/worGAL4tshGAL80_GFP.tif]

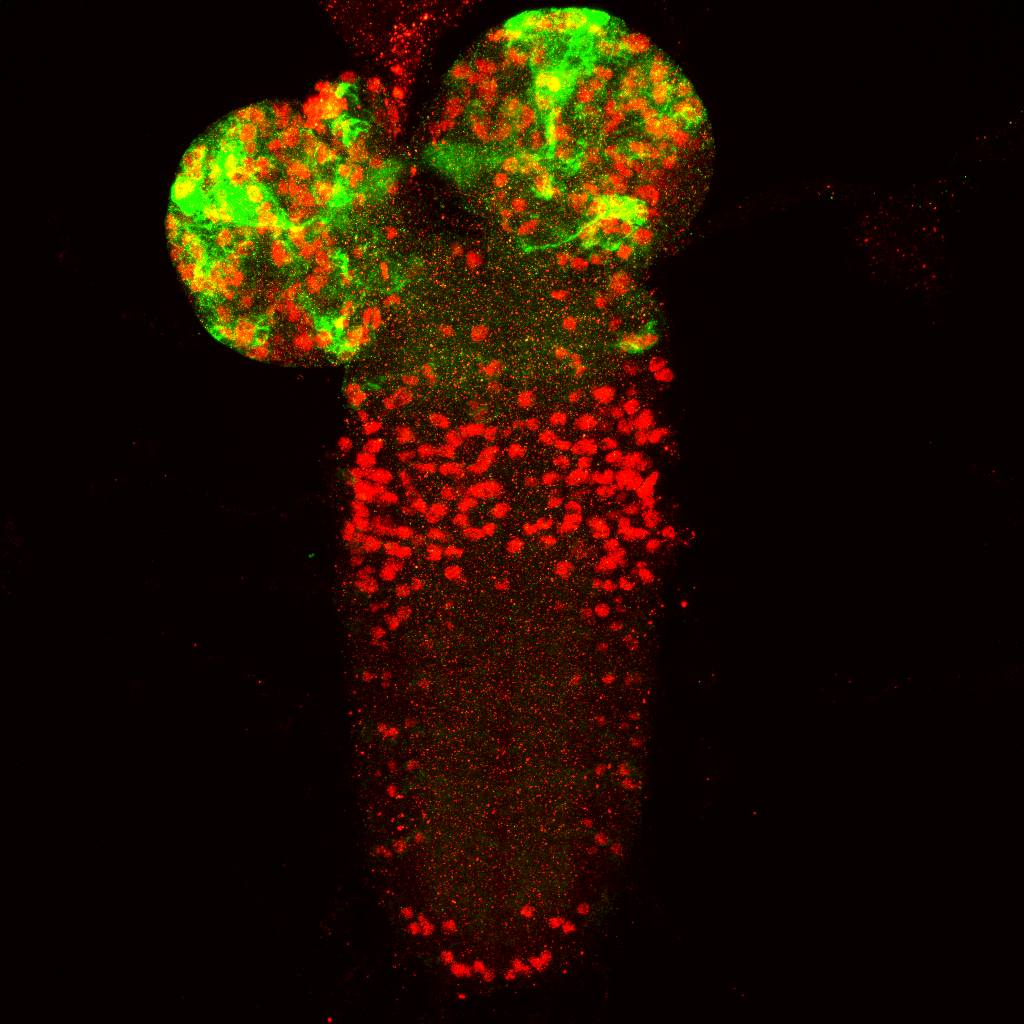

Supplement: Supplementary file 3 — Source data Fig. 2 [file 44318_2026_775_MOESM3_ESM.zip › Figure 2/2A/worGAL4tshGAL80_GFPDpn.tif]

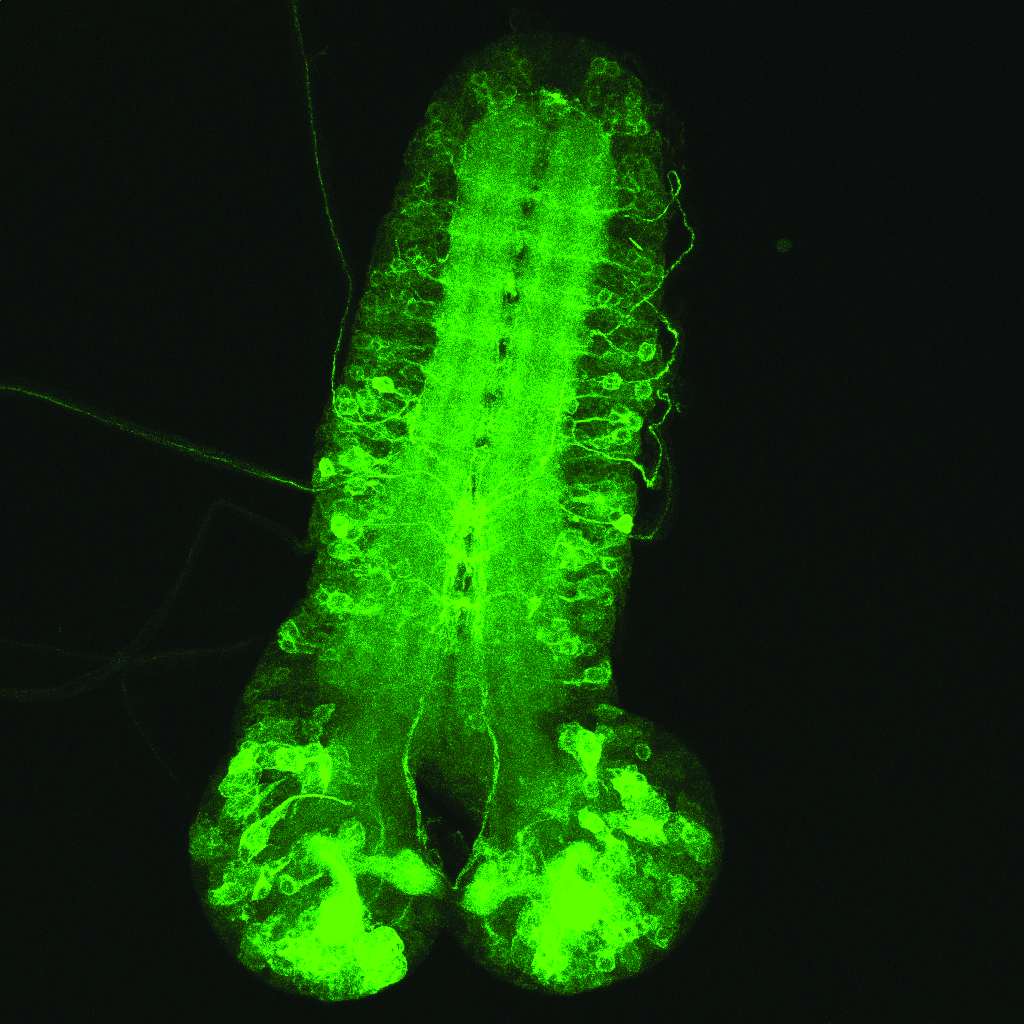

Supplement: Supplementary file 3 — Source data Fig. 2 [file 44318_2026_775_MOESM3_ESM.zip › Figure 2/2A/worGAL4GFP.tif]

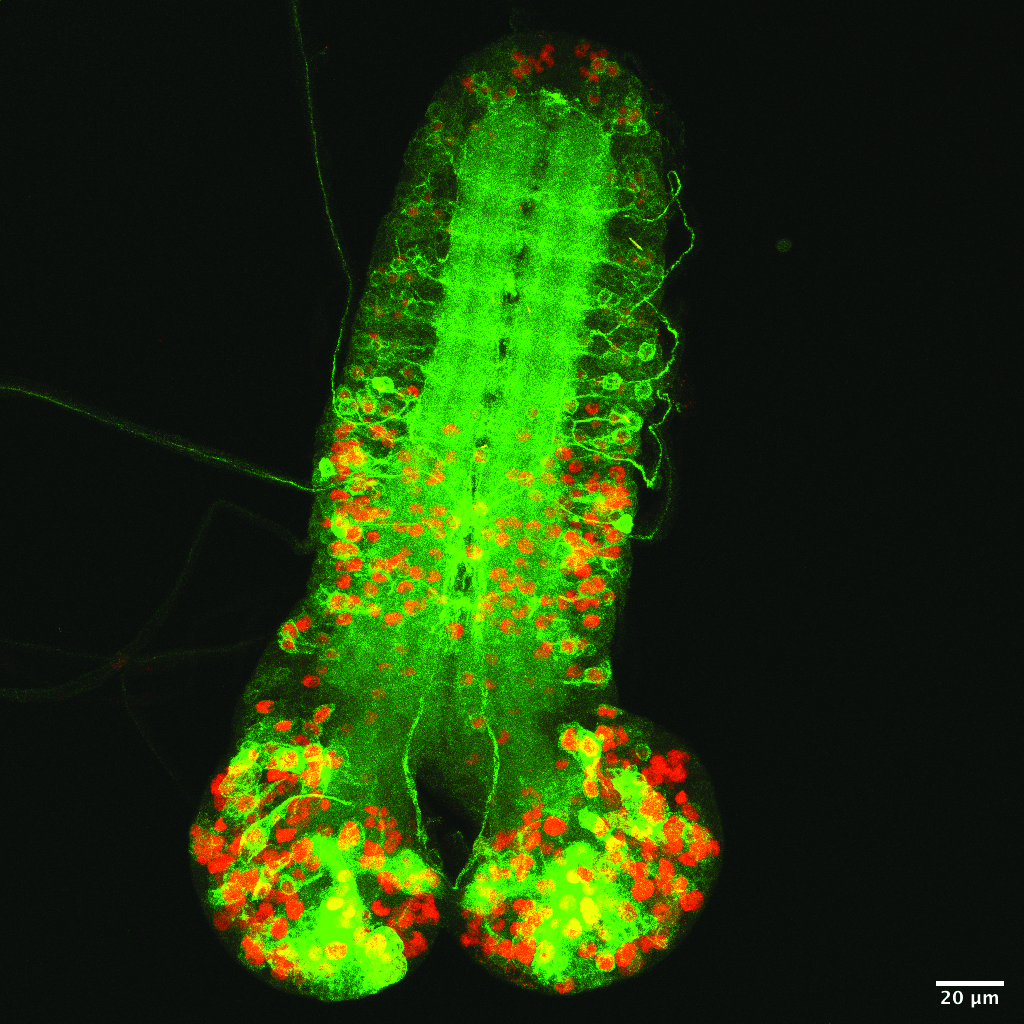

Supplement: Supplementary file 3 — Source data Fig. 2 [file 44318_2026_775_MOESM3_ESM.zip › Figure 2/2A/worGAL4GFPDpn.tif]

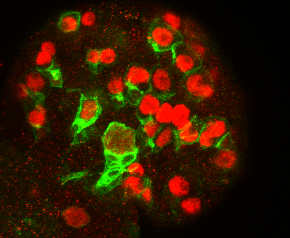

Supplement: Supplementary file 4 — Source data Fig. 3 [file 44318_2026_775_MOESM4_ESM.zip › Figure 3/3A/DpnGFP_BL.tif]

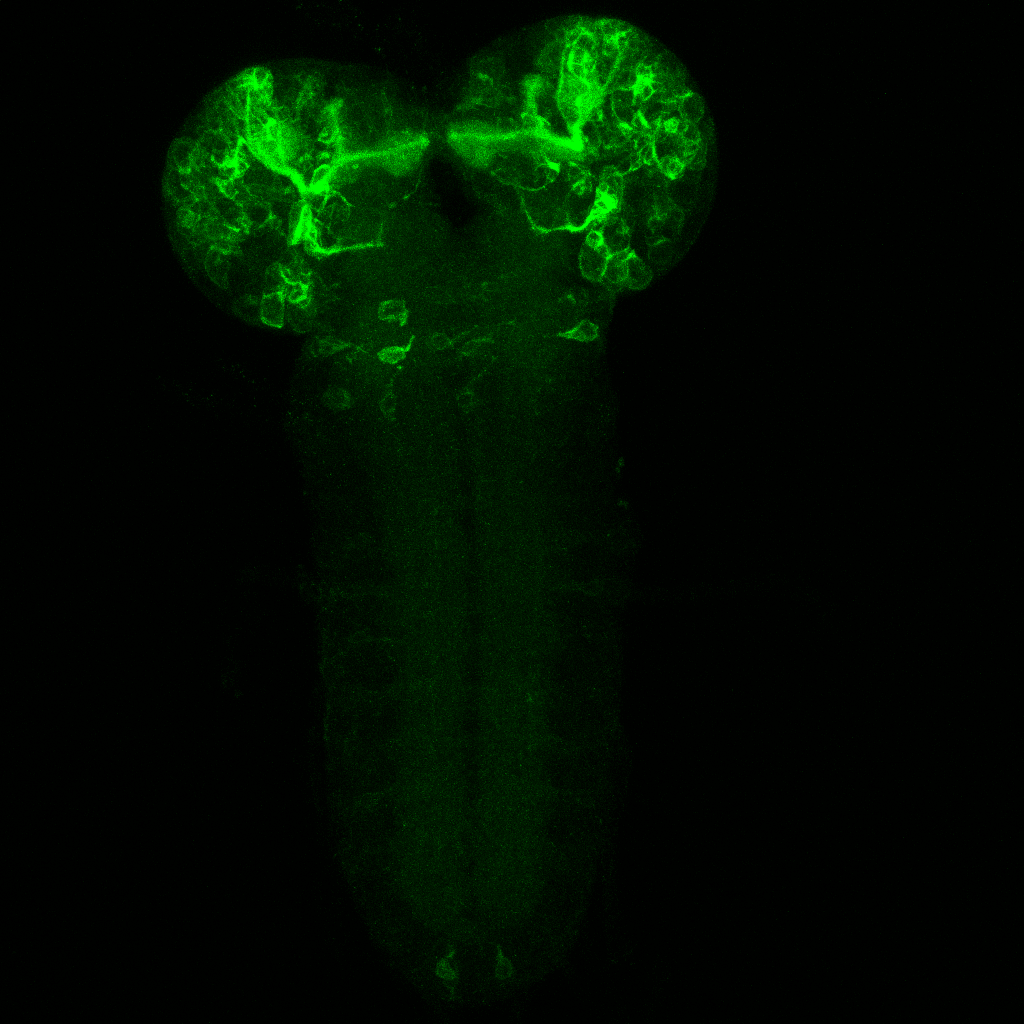

Supplement: Supplementary file 4 — Source data Fig. 3 [file 44318_2026_775_MOESM4_ESM.zip › Figure 3/3A/GFP_Kir21.tif]

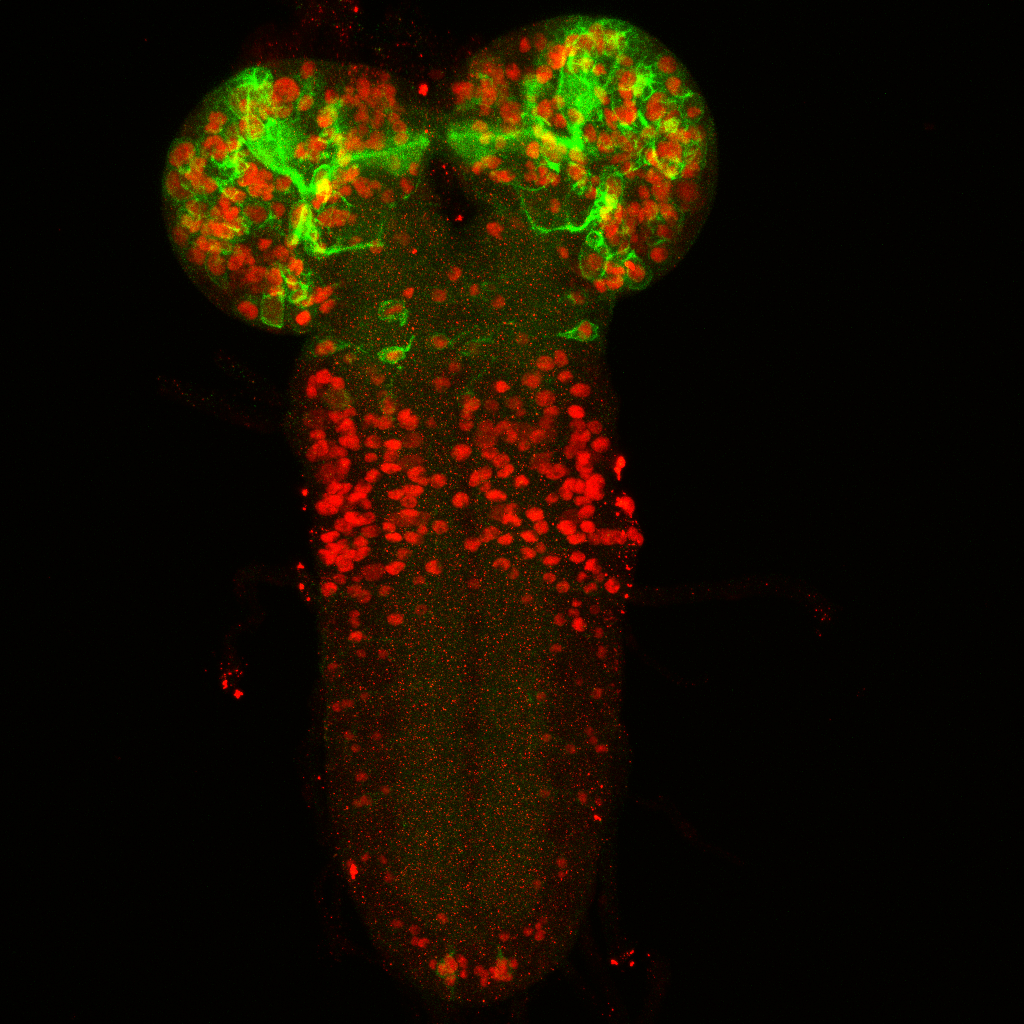

Supplement: Supplementary file 4 — Source data Fig. 3 [file 44318_2026_775_MOESM4_ESM.zip › Figure 3/3A/DpnGFP_Kir21.tif]

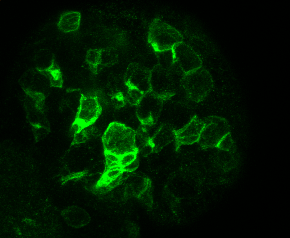

Supplement: Supplementary file 4 — Source data Fig. 3 [file 44318_2026_775_MOESM4_ESM.zip › Figure 3/3A/GFP_BL.tif .tif]

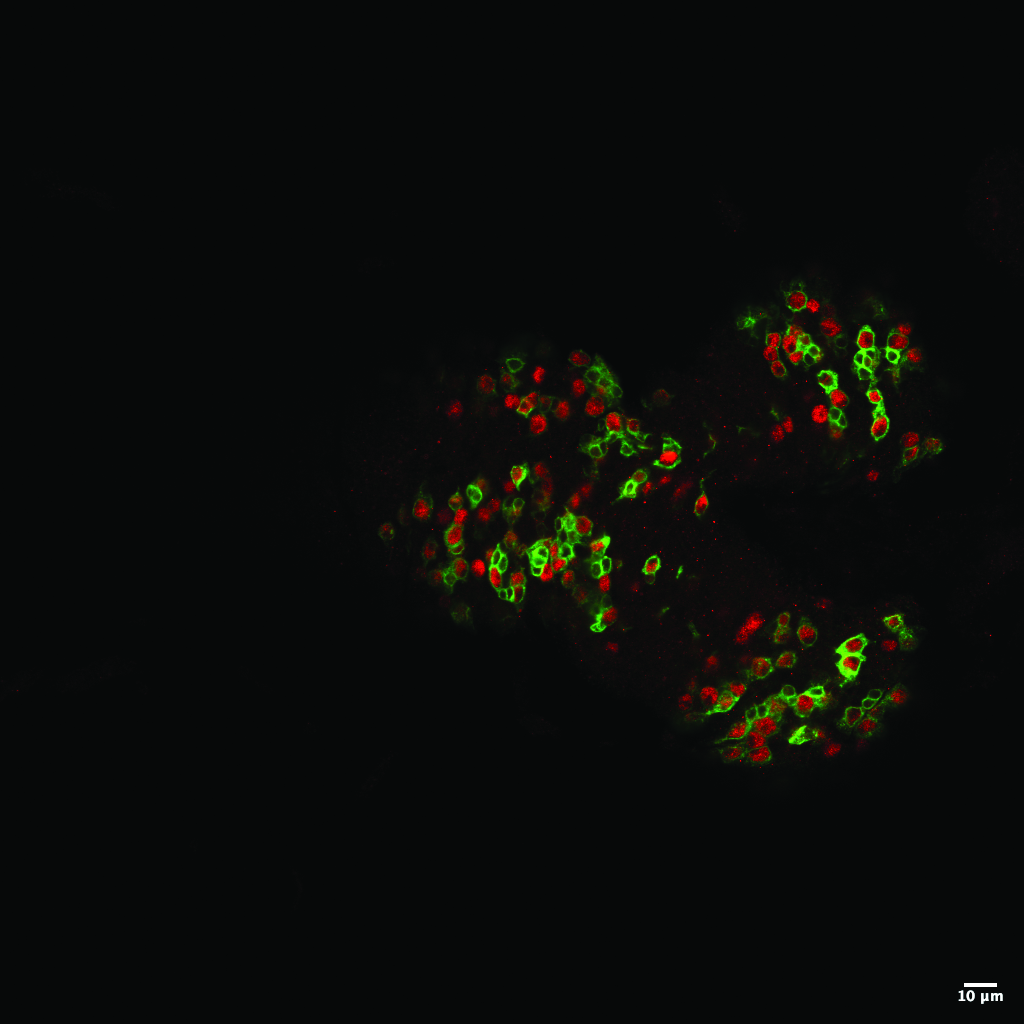

Supplement: Supplementary file 5 — Source data Fig. 4 [file 44318_2026_775_MOESM5_ESM.zip › Figure 4/4A/Reactivated NSCs.tif]

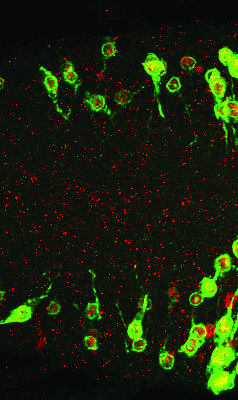

Supplement: Supplementary file 5 — Source data Fig. 4 [file 44318_2026_775_MOESM5_ESM.zip › Figure 4/4A/QuiescentNSCs.tif]

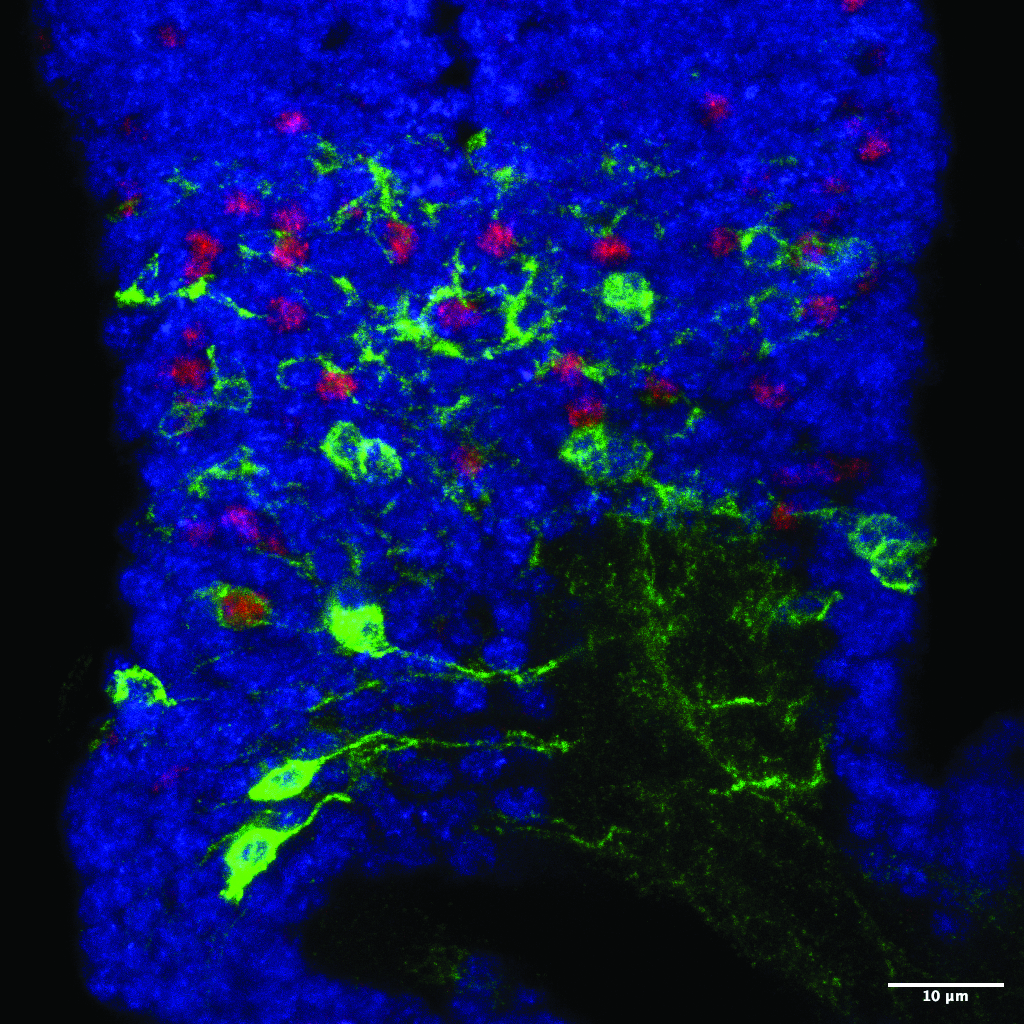

Supplement: Supplementary file 5 — Source data Fig. 4 [file 44318_2026_775_MOESM5_ESM.zip › Figure 4/4A/Neurons_NSCs.tif]

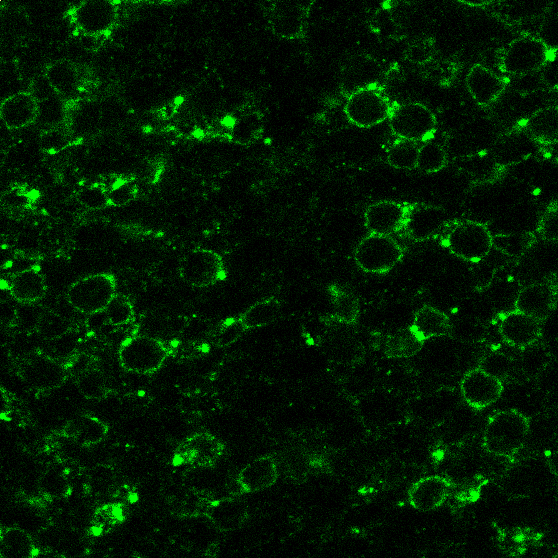

Supplement: Supplementary file 6 — Source data Fig. 5 [file 44318_2026_775_MOESM6_ESM.zip › Figure 5/5C/hig_0hrs.tif]

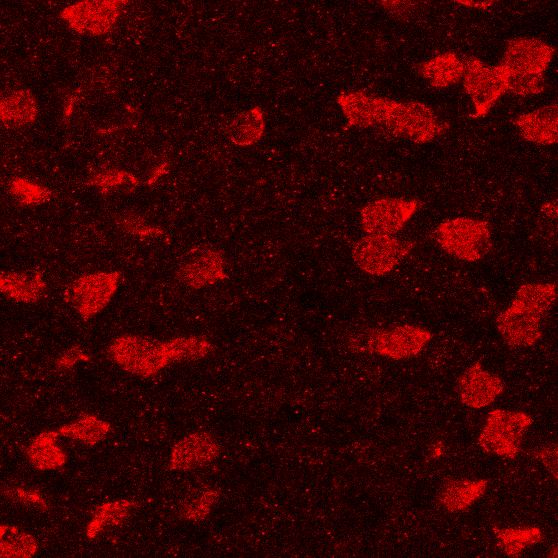

Supplement: Supplementary file 6 — Source data Fig. 5 [file 44318_2026_775_MOESM6_ESM.zip › Figure 5/5C/dpn_0hrs.tif]

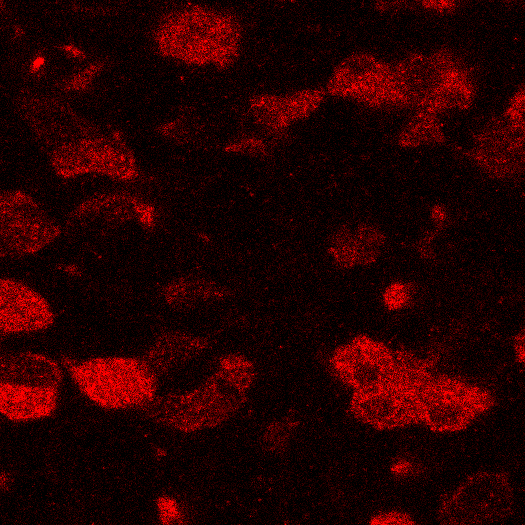

Supplement: Supplementary file 6 — Source data Fig. 5 [file 44318_2026_775_MOESM6_ESM.zip › Figure 5/5C/dpn_24hrs.tif]

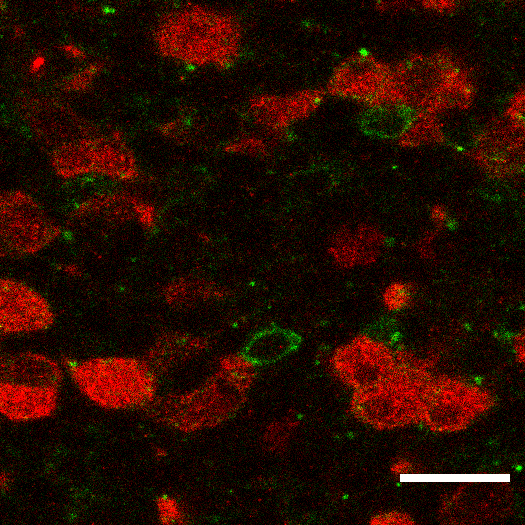

Supplement: Supplementary file 6 — Source data Fig. 5 [file 44318_2026_775_MOESM6_ESM.zip › Figure 5/5C/dpnhig_24hrs.tif]

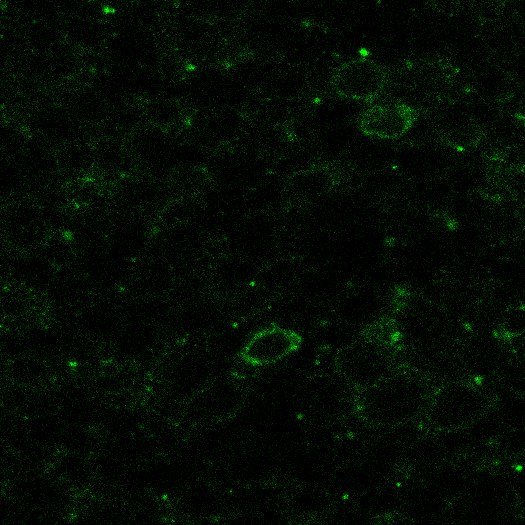

Supplement: Supplementary file 6 — Source data Fig. 5 [file 44318_2026_775_MOESM6_ESM.zip › Figure 5/5C/hig_24hrs.tif]

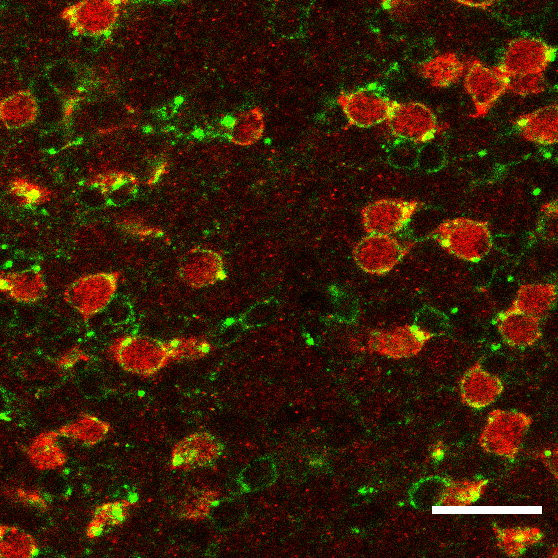

Supplement: Supplementary file 6 — Source data Fig. 5 [file 44318_2026_775_MOESM6_ESM.zip › Figure 5/5C/dpnhig_0hrs.tif]

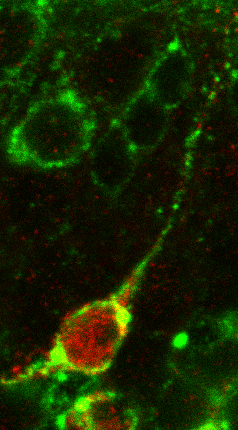

Supplement: Supplementary file 6 — Source data Fig. 5 [file 44318_2026_775_MOESM6_ESM.zip › Figure 5/5D/zoom Hig NSC.tif]

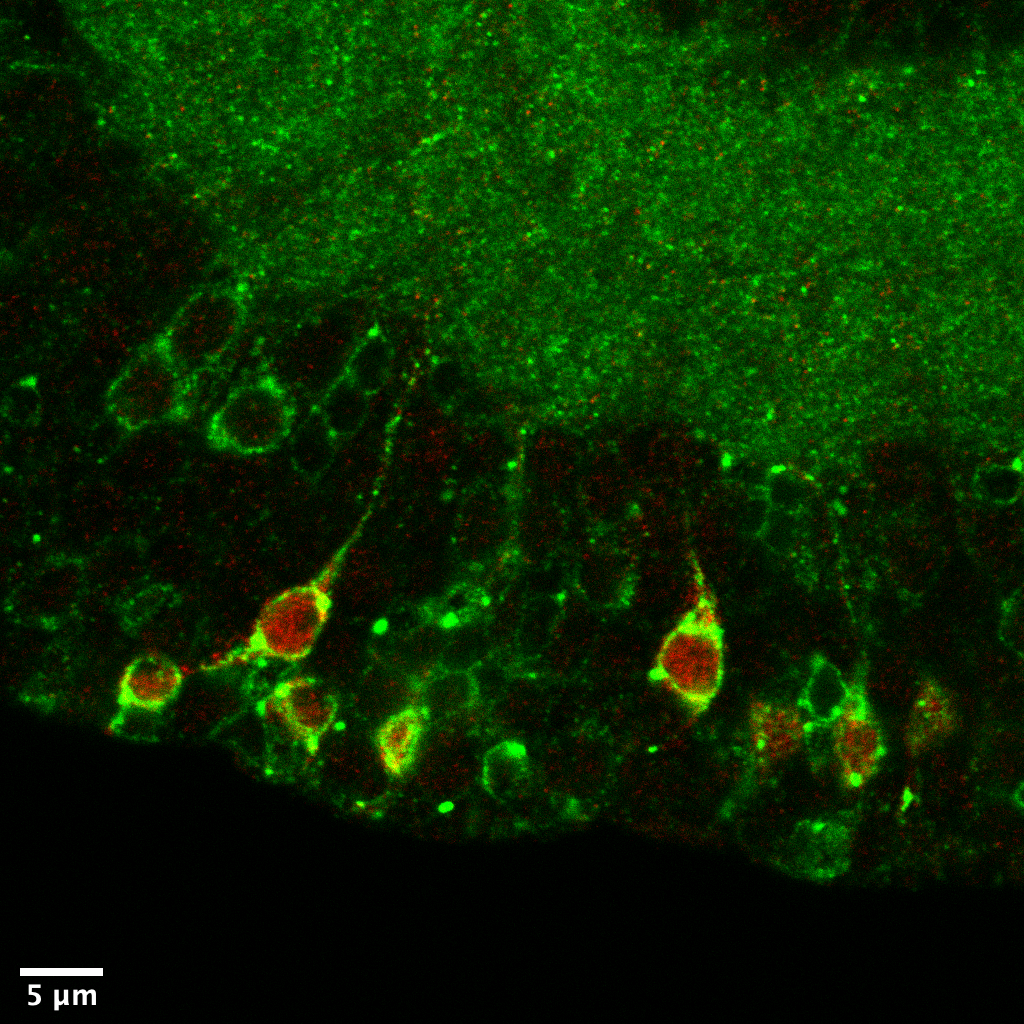

Supplement: Supplementary file 6 — Source data Fig. 5 [file 44318_2026_775_MOESM6_ESM.zip › Figure 5/5D/Hig NSCs.tif]

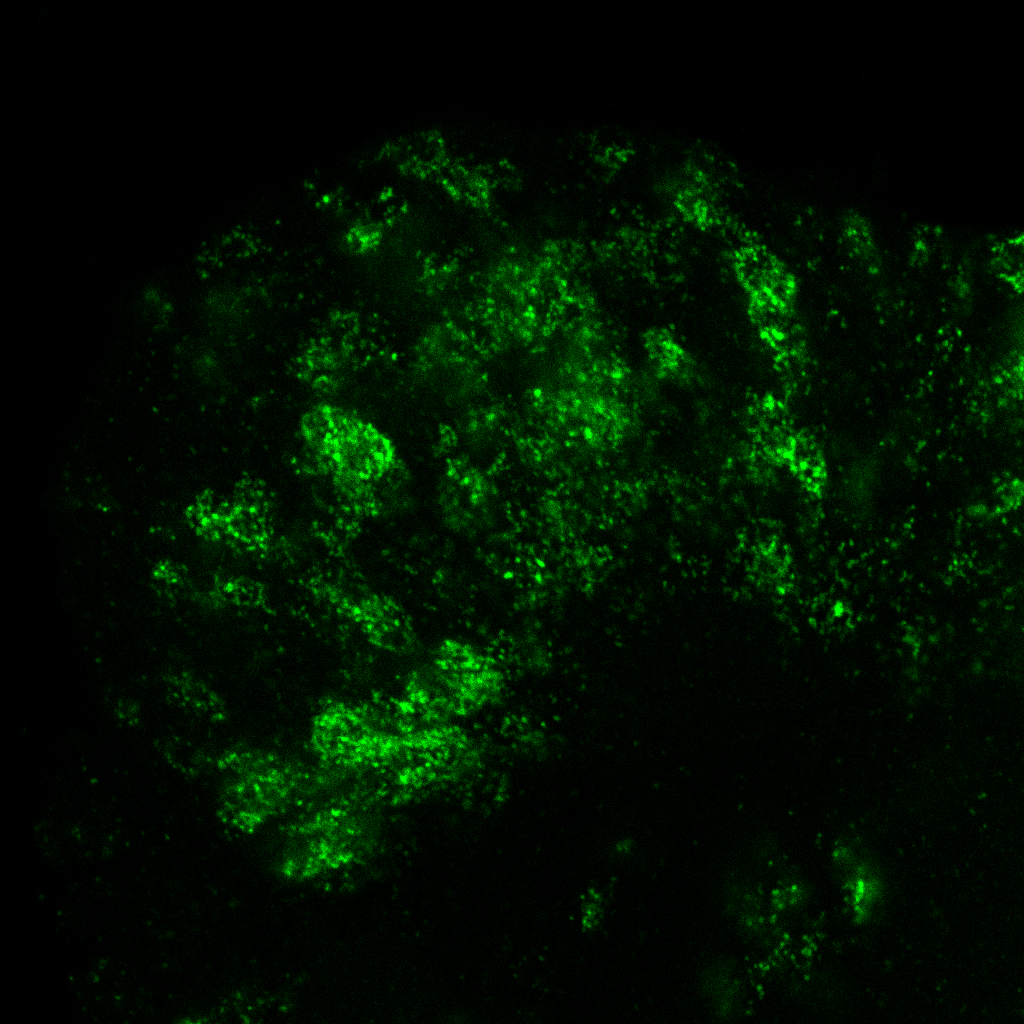

Supplement: Supplementary file 6 — Source data Fig. 5 [file 44318_2026_775_MOESM6_ESM.zip › Figure 5/5B/hig_0hrs.tif]

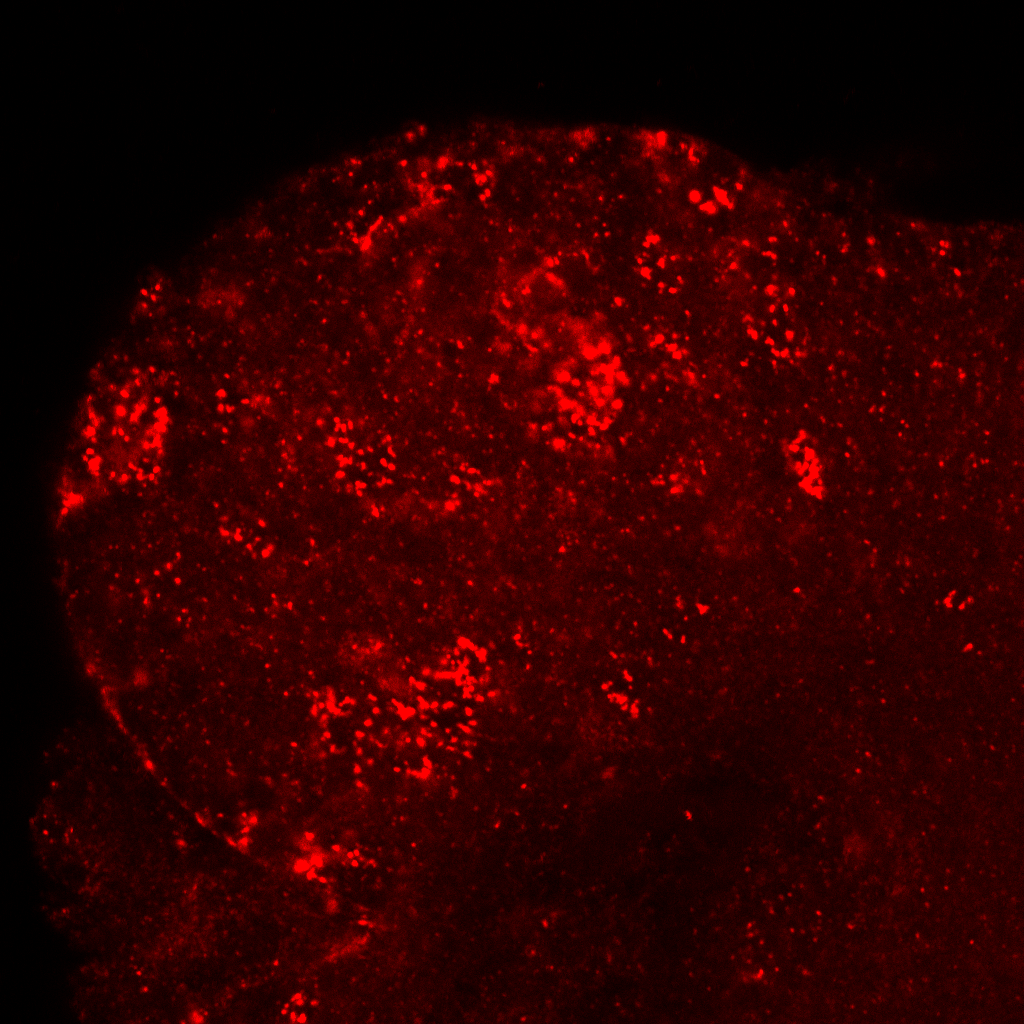

Supplement: Supplementary file 6 — Source data Fig. 5 [file 44318_2026_775_MOESM6_ESM.zip › Figure 5/5B/dpn0hrs.tif]

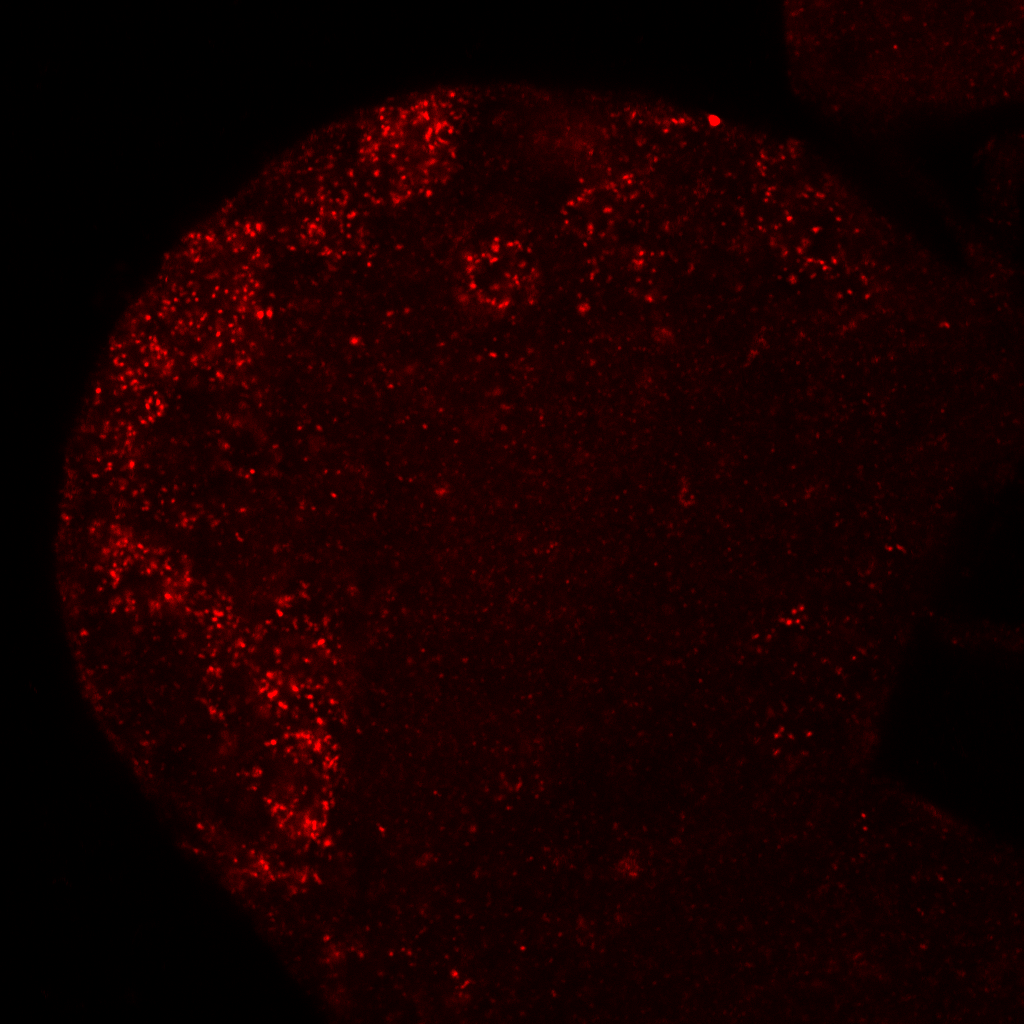

Supplement: Supplementary file 6 — Source data Fig. 5 [file 44318_2026_775_MOESM6_ESM.zip › Figure 5/5B/dpn_24hrs.tif]

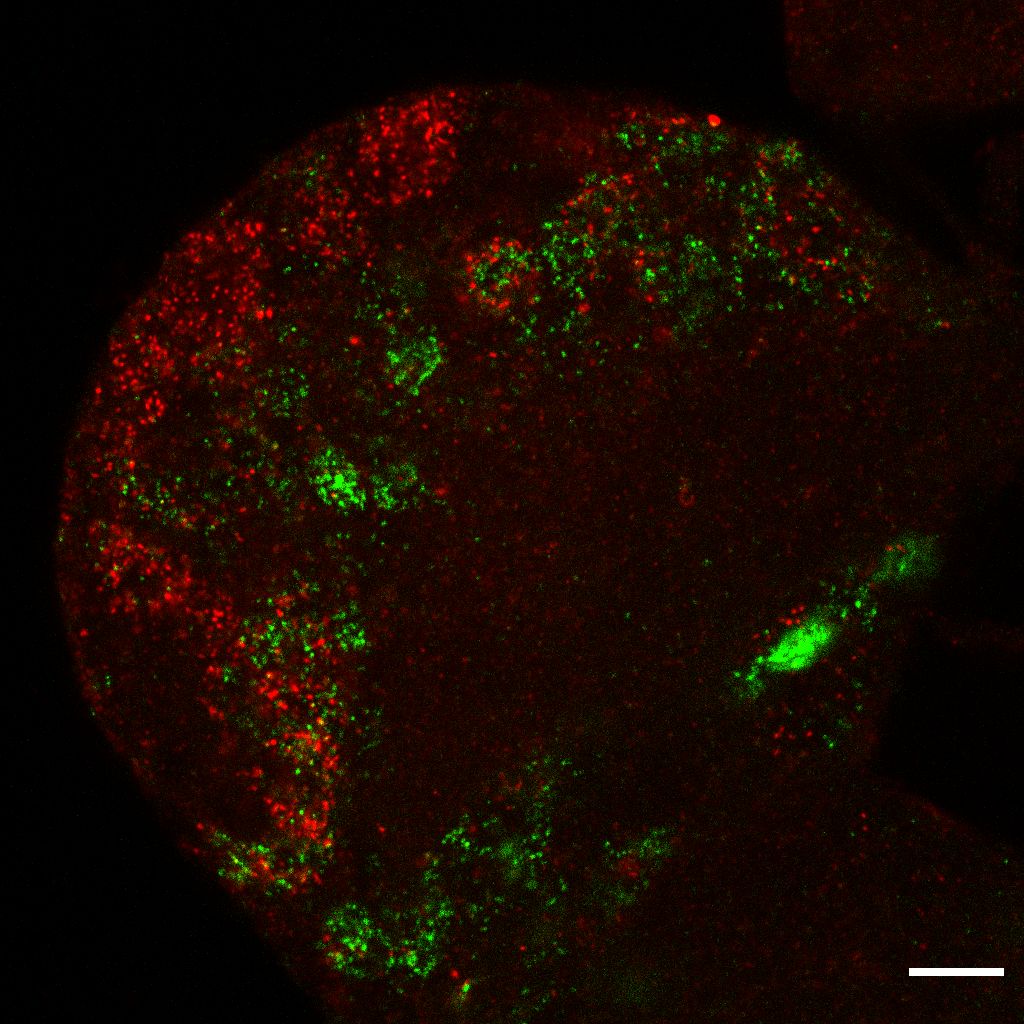

Supplement: Supplementary file 6 — Source data Fig. 5 [file 44318_2026_775_MOESM6_ESM.zip › Figure 5/5B/dpnhig_24hrs.tif]

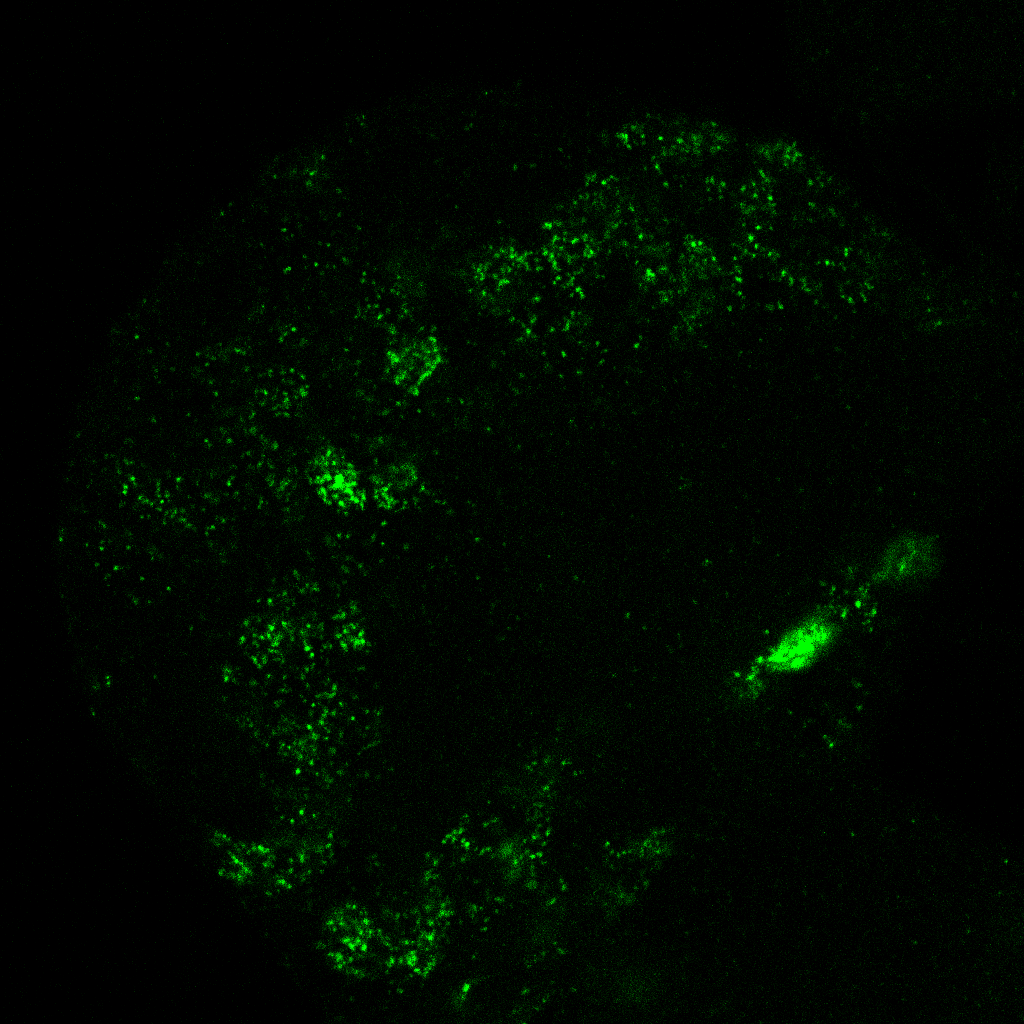

Supplement: Supplementary file 6 — Source data Fig. 5 [file 44318_2026_775_MOESM6_ESM.zip › Figure 5/5B/hig_24hrs.tif]

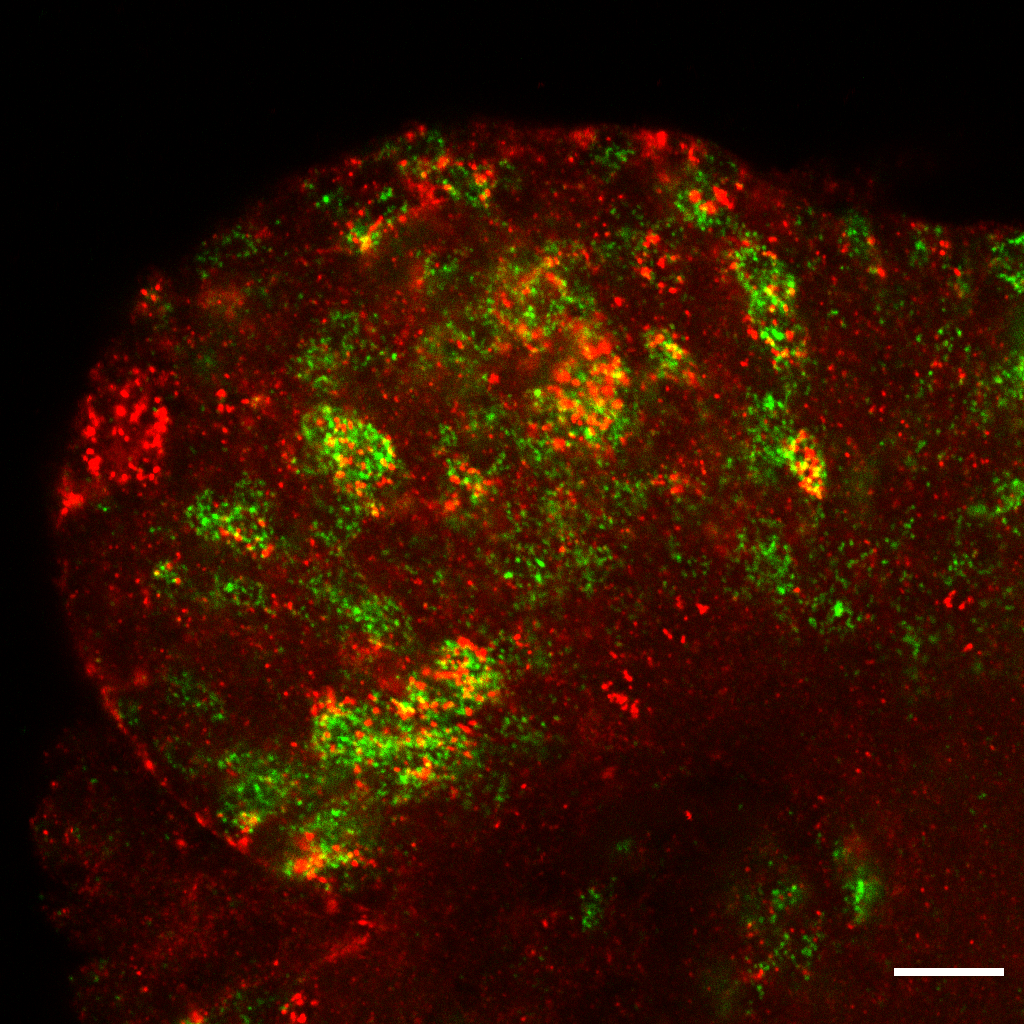

Supplement: Supplementary file 6 — Source data Fig. 5 [file 44318_2026_775_MOESM6_ESM.zip › Figure 5/5B/dpnhig_0hrs.tif]

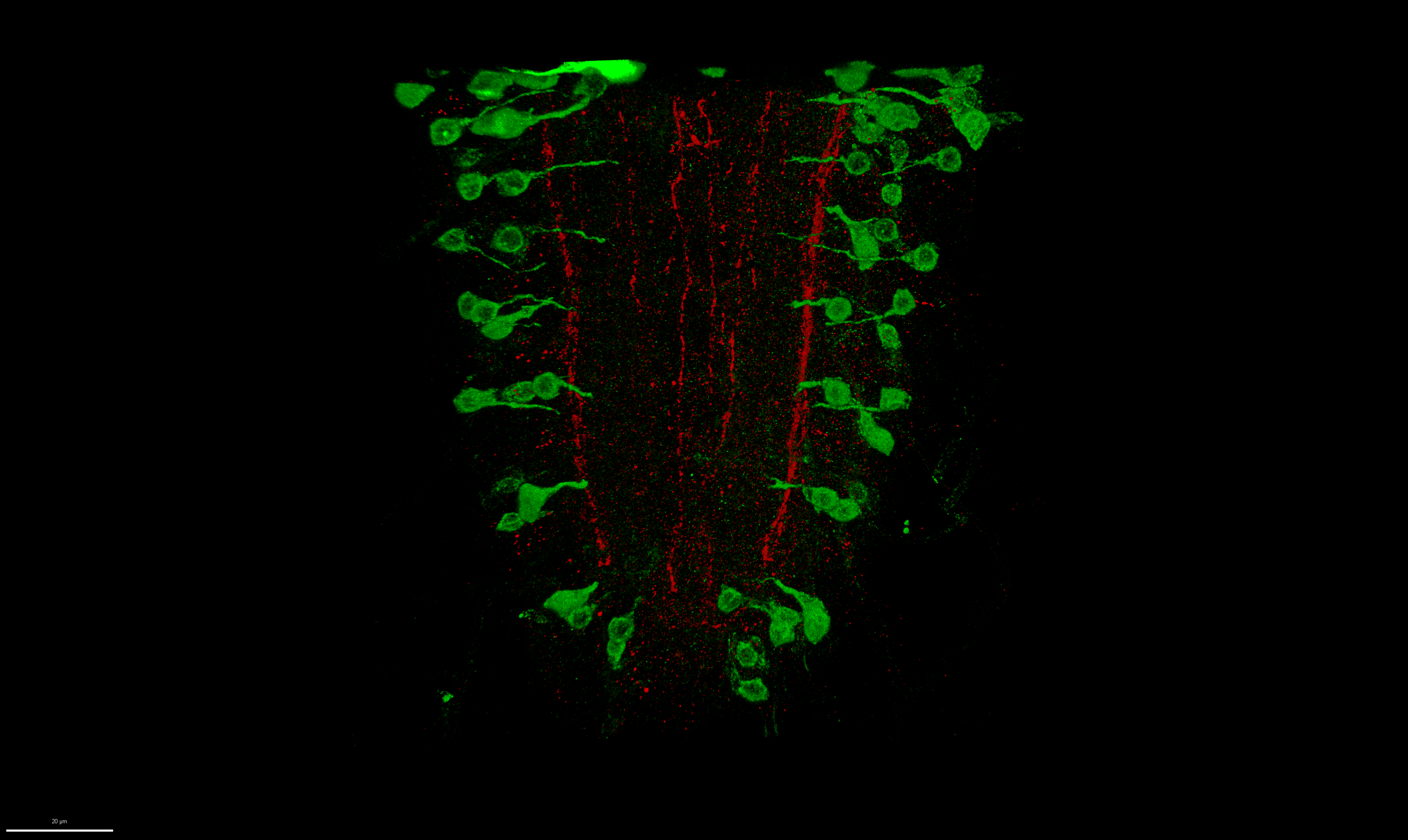

Supplement: Supplementary file 7 — Source data Fig. 6 [file 44318_2026_775_MOESM7_ESM.zip › Figure 6/6A/3D.tif]

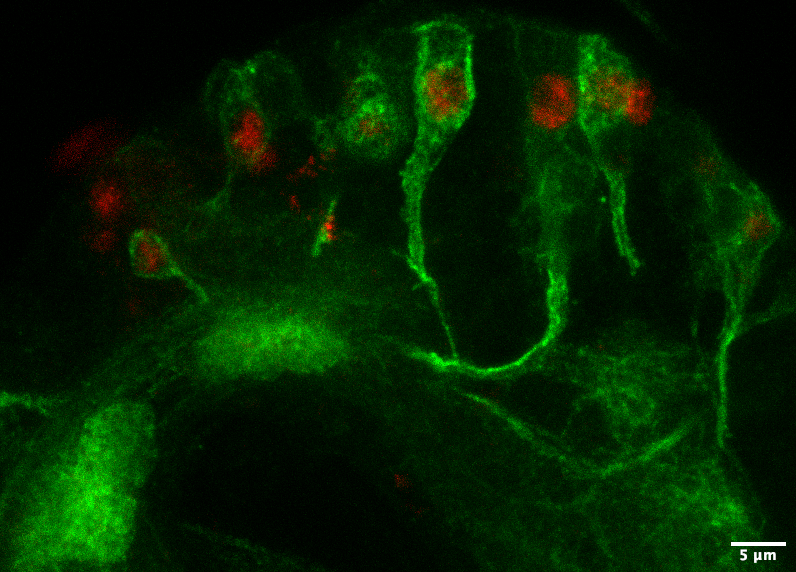

Supplement: Supplementary file 7 — Source data Fig. 6 [file 44318_2026_775_MOESM7_ESM.zip › Figure 6/6B/6B_BL.tif]

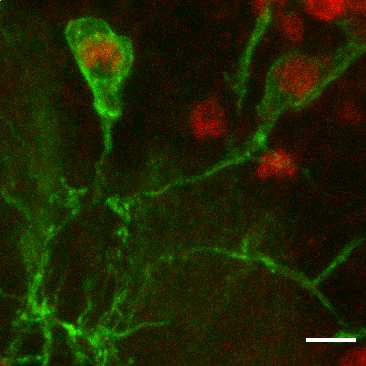

Supplement: Supplementary file 7 — Source data Fig. 6 [file 44318_2026_775_MOESM7_ESM.zip › Figure 6/6B/6B_VNC.tif]

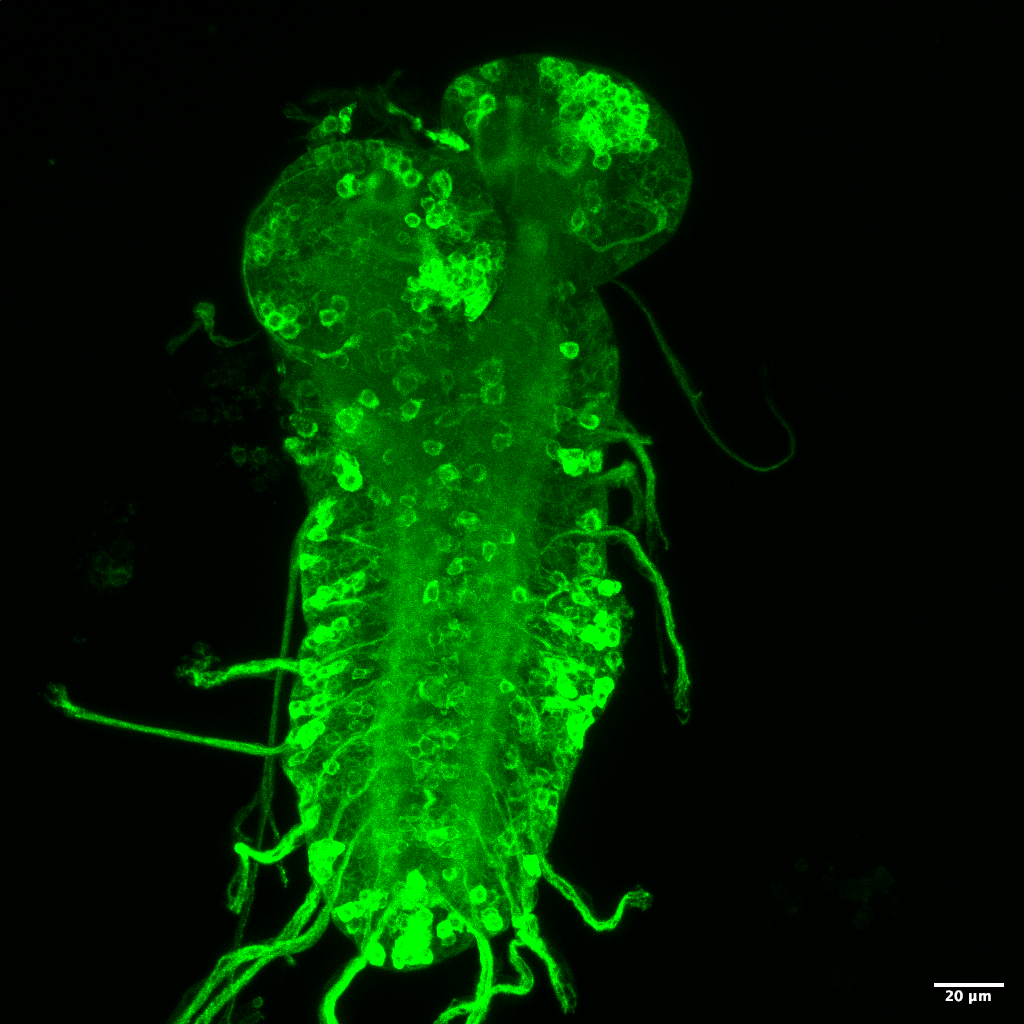

Supplement: Supplementary file 7 — Source data Fig. 6 [file 44318_2026_775_MOESM7_ESM.zip › Figure 6/6C/elavGAL4.tif]

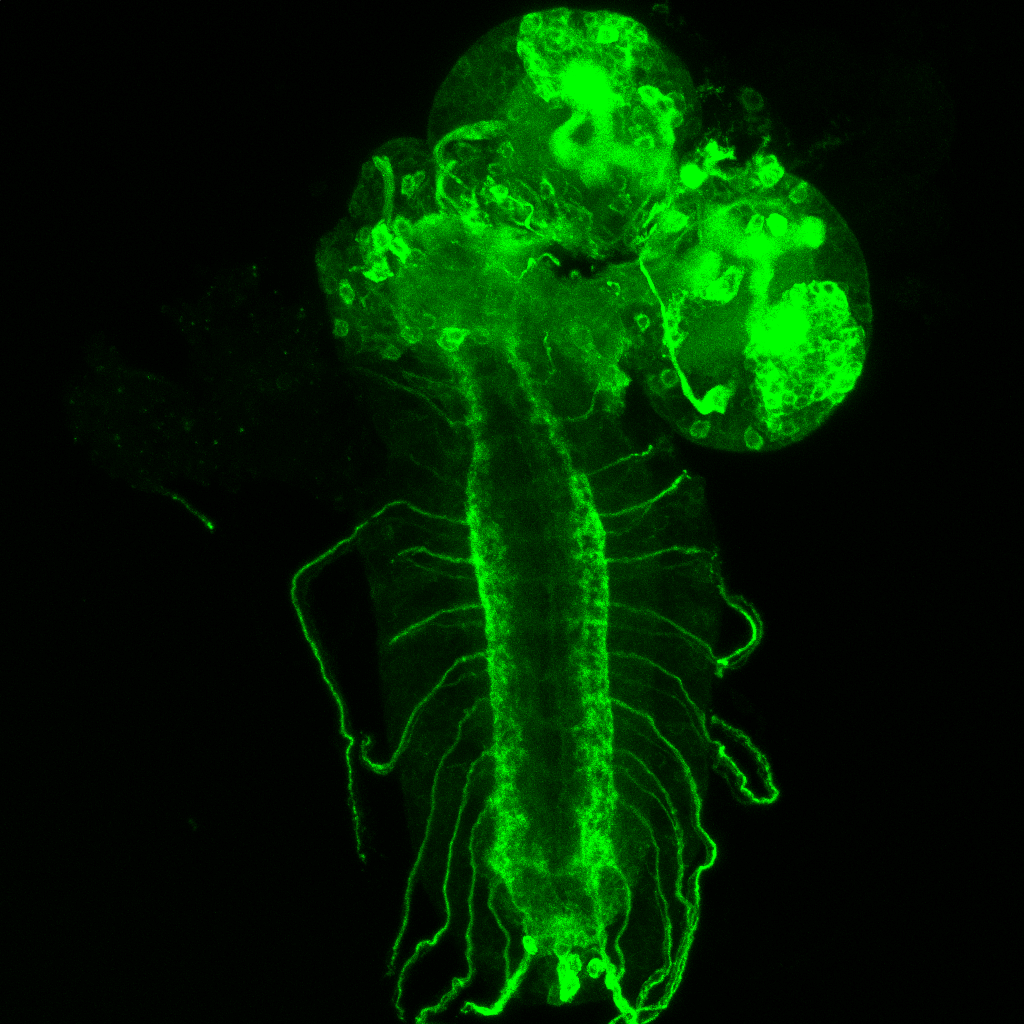

Supplement: Supplementary file 7 — Source data Fig. 6 [file 44318_2026_775_MOESM7_ESM.zip › Figure 6/6C/elavGAL4tshGAL80.tif]

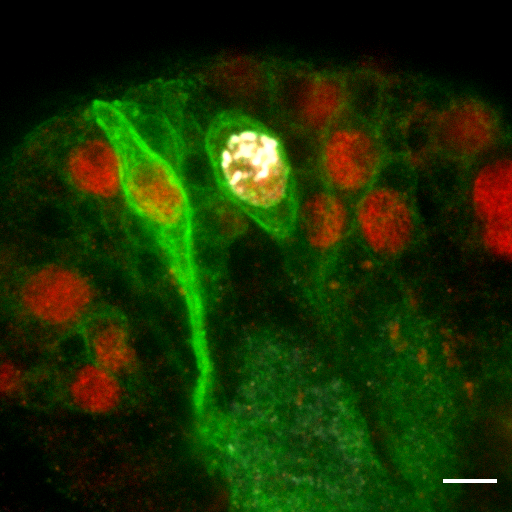

Supplement: Supplementary file 8 — Source data Fig. 7 [file 44318_2026_775_MOESM8_ESM.zip › Figure 7/7A/7A.tif]

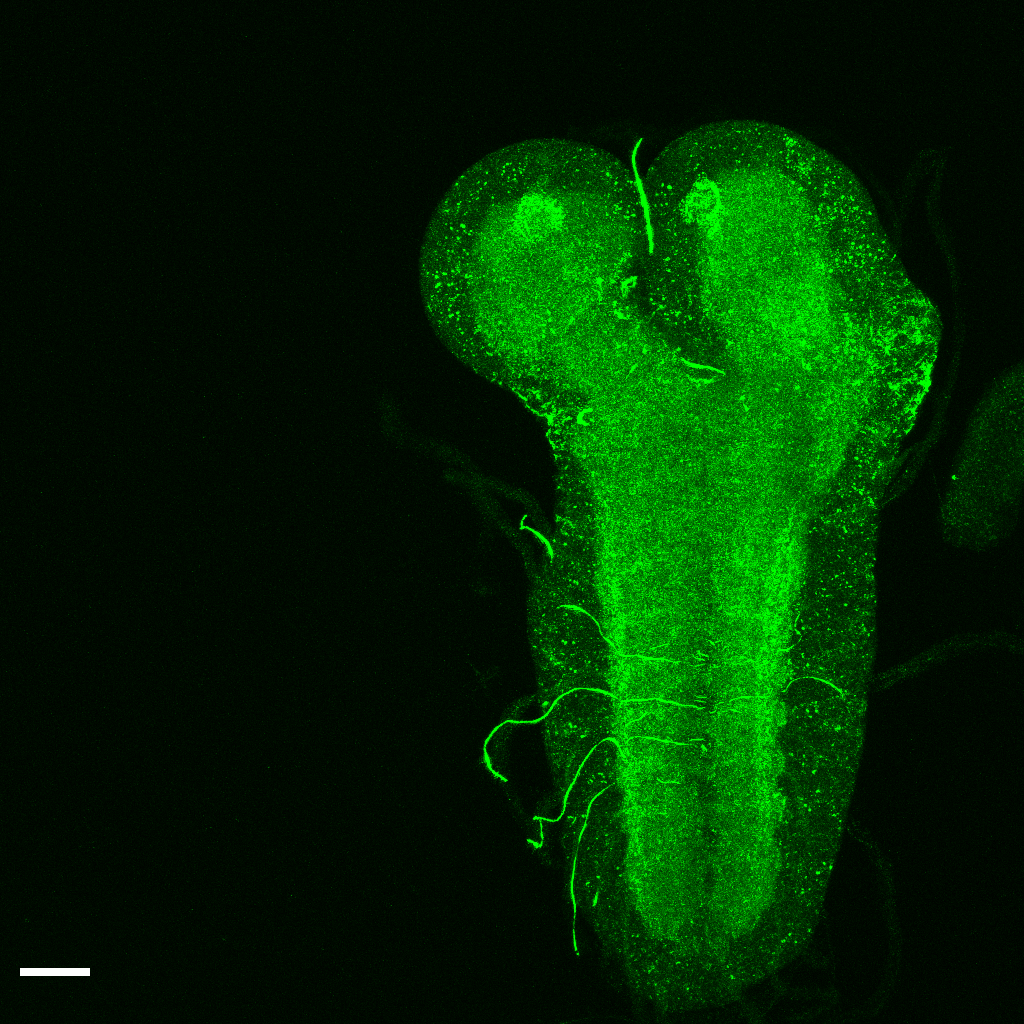

Supplement: Supplementary file 9 — Figure EV3 Source Data [file 44318_2026_775_MOESM9_ESM.zip › Figure EV3/EV3A/EV3_control.tif]

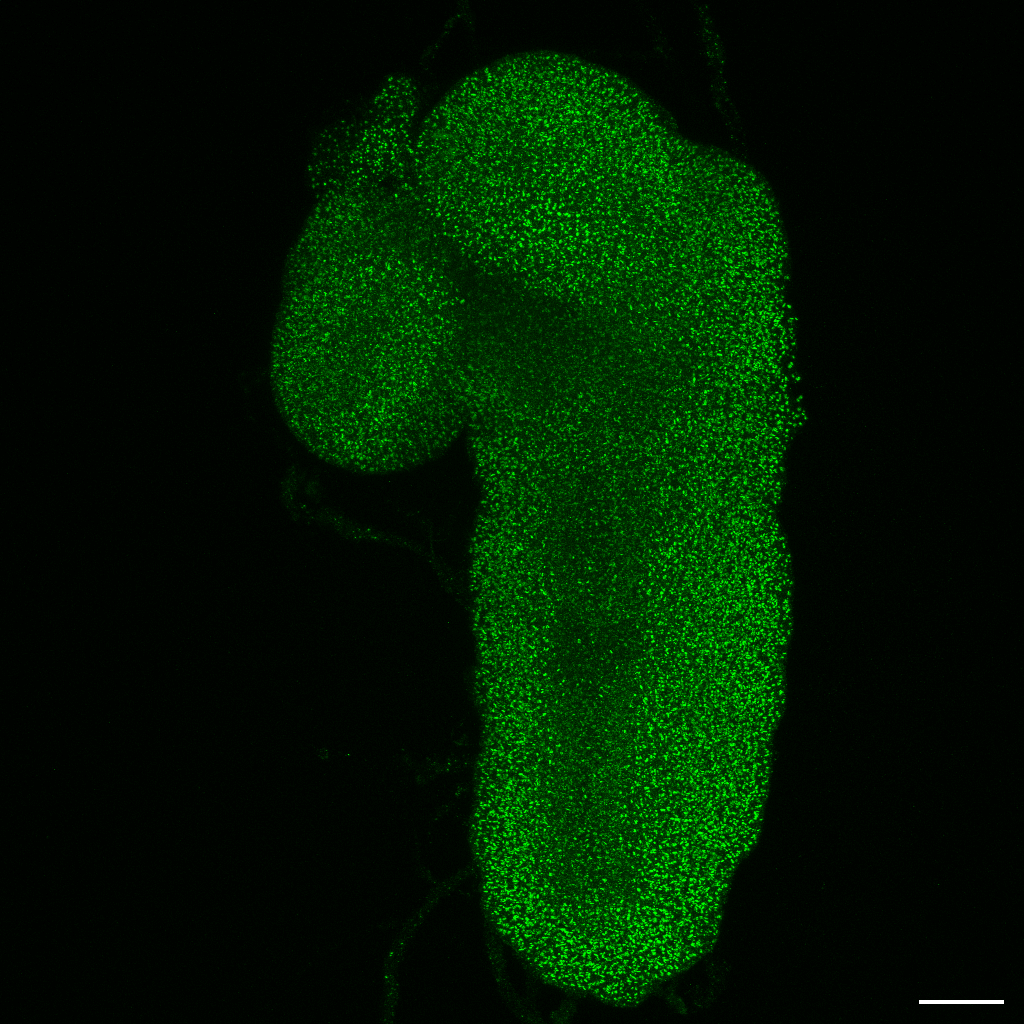

Supplement: Supplementary file 9 — Figure EV3 Source Data [file 44318_2026_775_MOESM9_ESM.zip › Figure EV3/EV3A/EB3_higRNAi.tif]

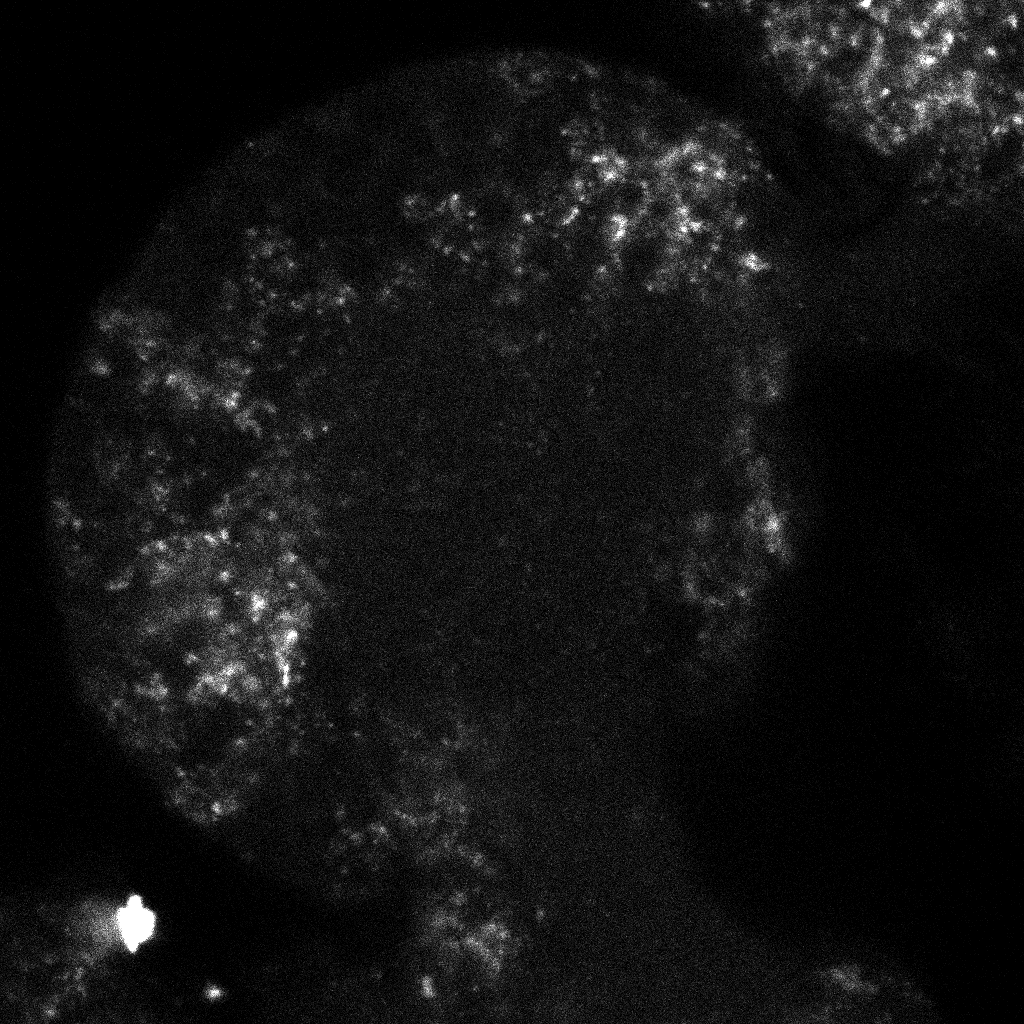

Supplement: Supplementary file 9 — Figure EV3 Source Data [file 44318_2026_775_MOESM9_ESM.zip › Figure EV3/EV3B/Hig.tif]

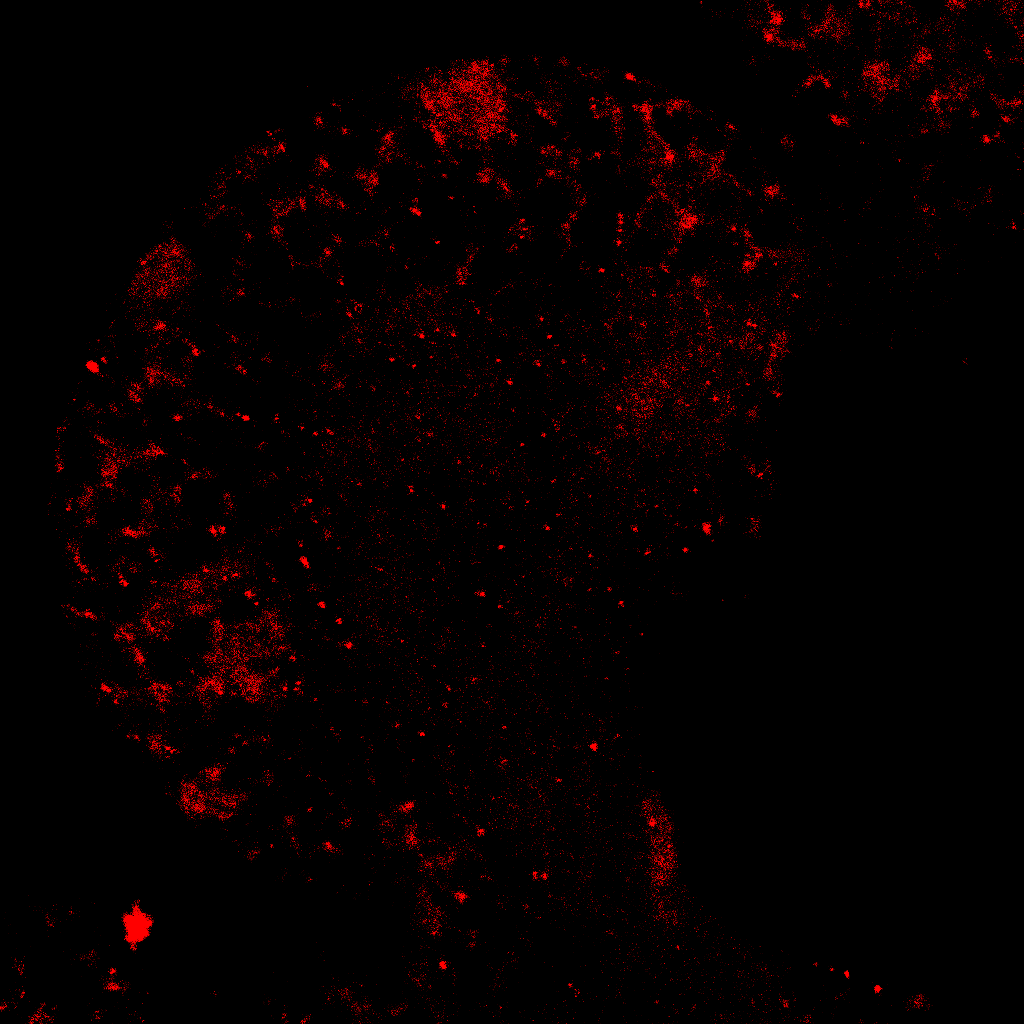

Supplement: Supplementary file 9 — Figure EV3 Source Data [file 44318_2026_775_MOESM9_ESM.zip › Figure EV3/EV3B/Dpn.tif]

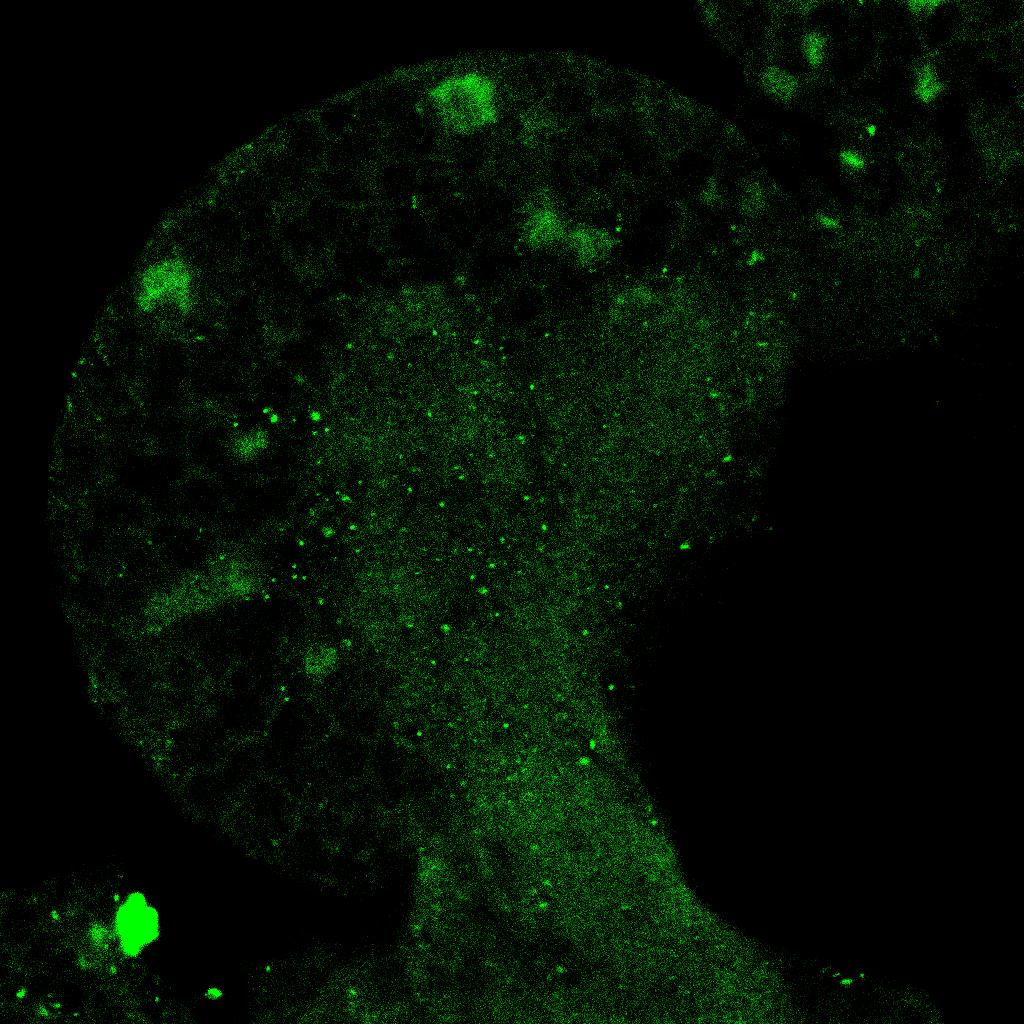

Supplement: Supplementary file 9 — Figure EV3 Source Data [file 44318_2026_775_MOESM9_ESM.zip › Figure EV3/EV3B/Rx.tif]

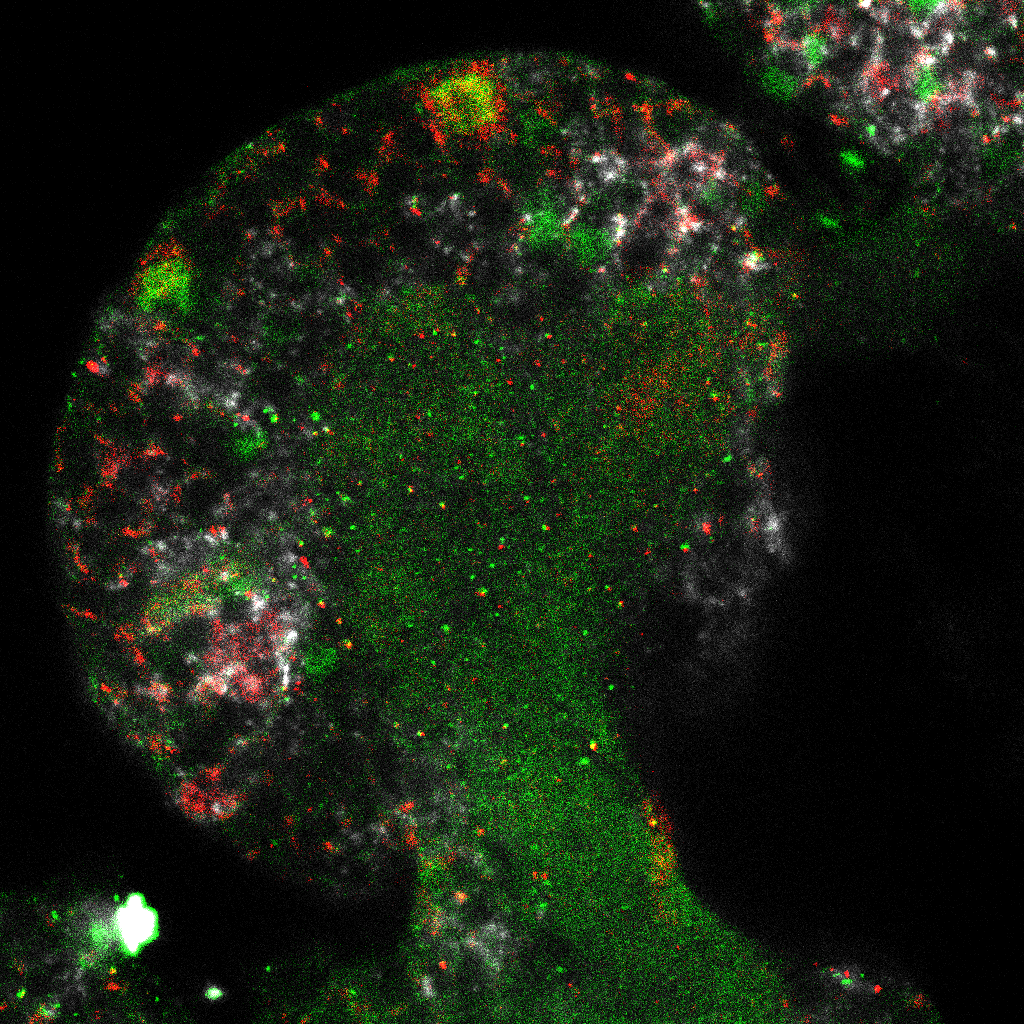

Supplement: Supplementary file 9 — Figure EV3 Source Data [file 44318_2026_775_MOESM9_ESM.zip › Figure EV3/EV3B/Merge.tif]

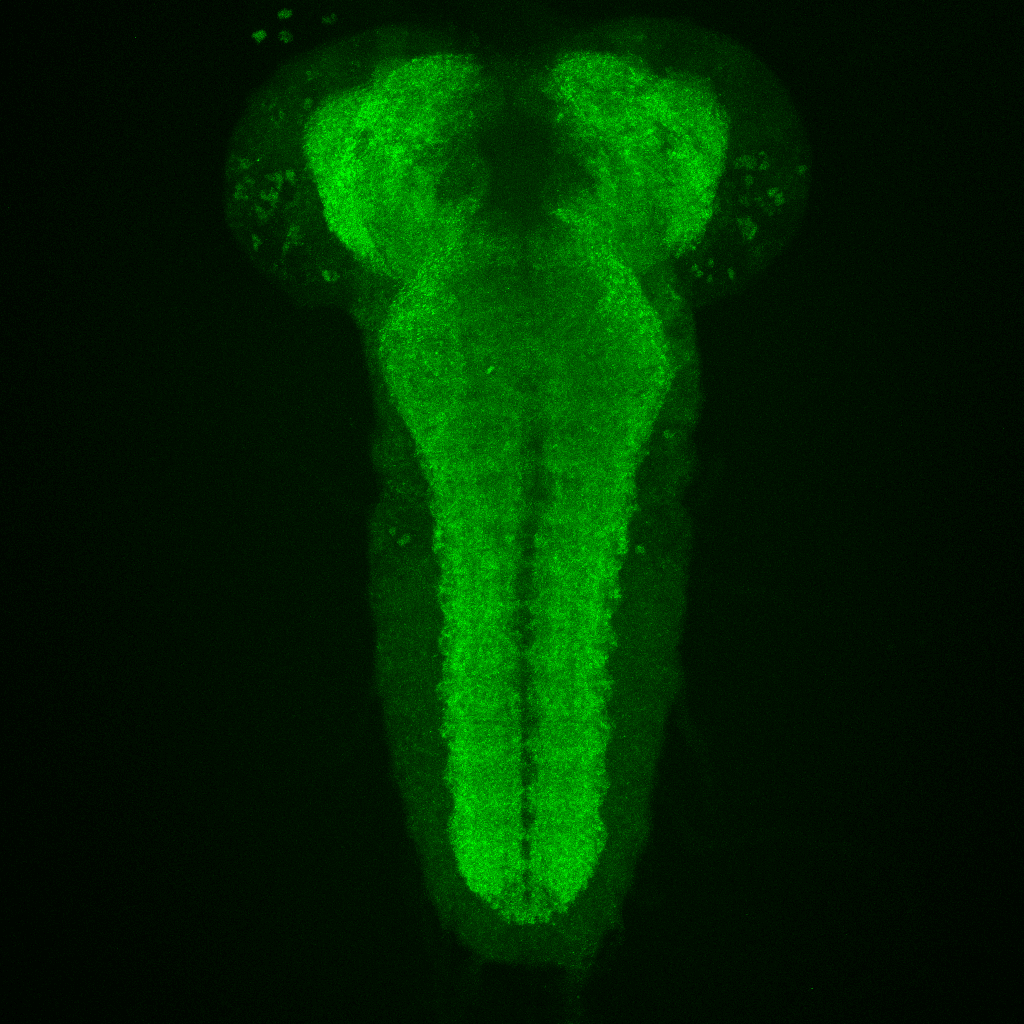

Supplement: Supplementary file 10 — Figure EV4 Source Data [file 44318_2026_775_MOESM10_ESM.zip › Figure EV4/EV4A/control.tif]

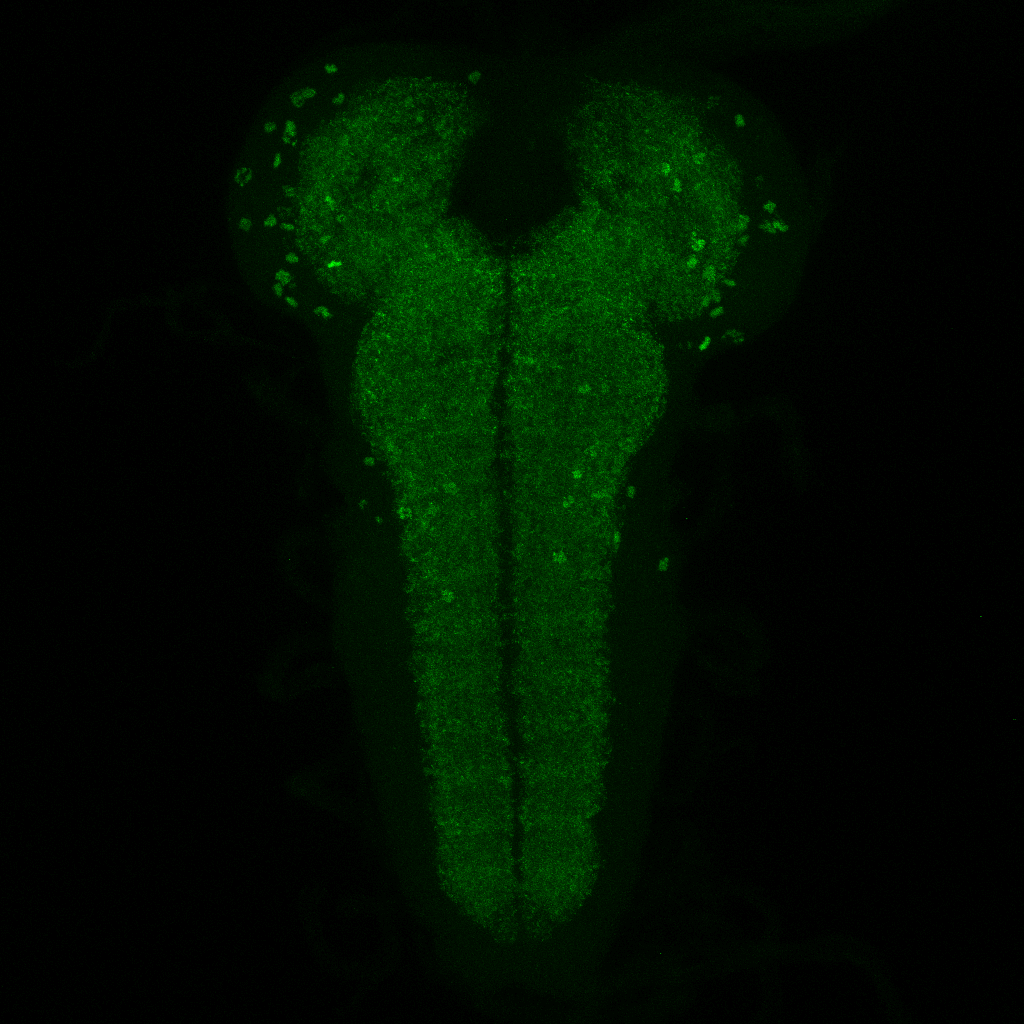

Supplement: Supplementary file 10 — Figure EV4 Source Data [file 44318_2026_775_MOESM10_ESM.zip › Figure EV4/EV4A/haspRNAi1.tif]

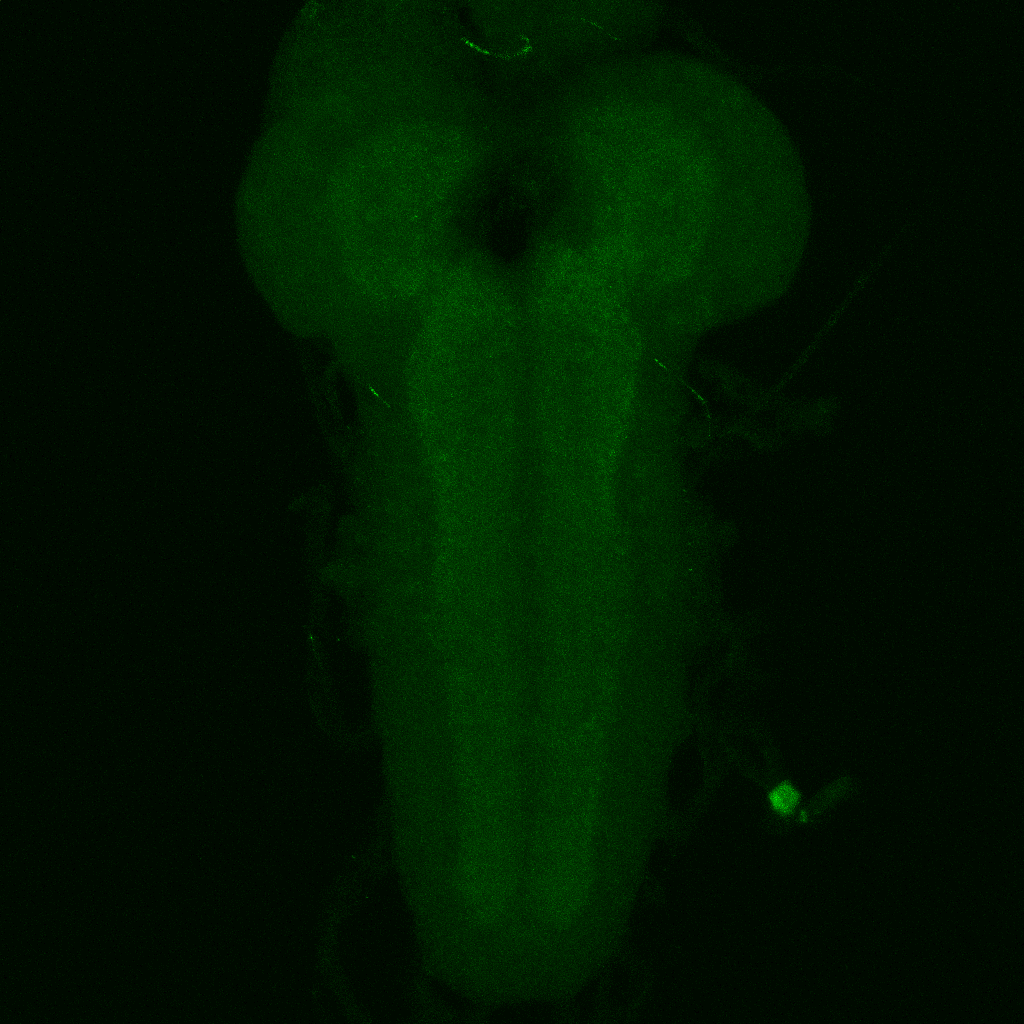

Supplement: Supplementary file 10 — Figure EV4 Source Data [file 44318_2026_775_MOESM10_ESM.zip › Figure EV4/EV4A/haspRNAi2.tif]

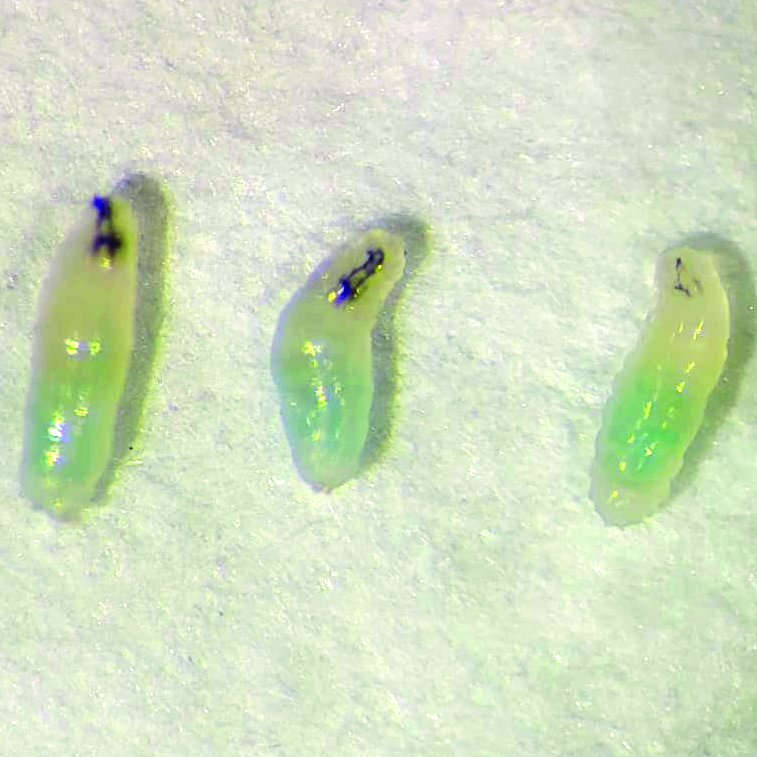

Supplement: Supplementary file 11 — Figure EV5 Source Data [file 44318_2026_775_MOESM11_ESM.zip › Figure EV5/EV5B/higRNAi.tif]

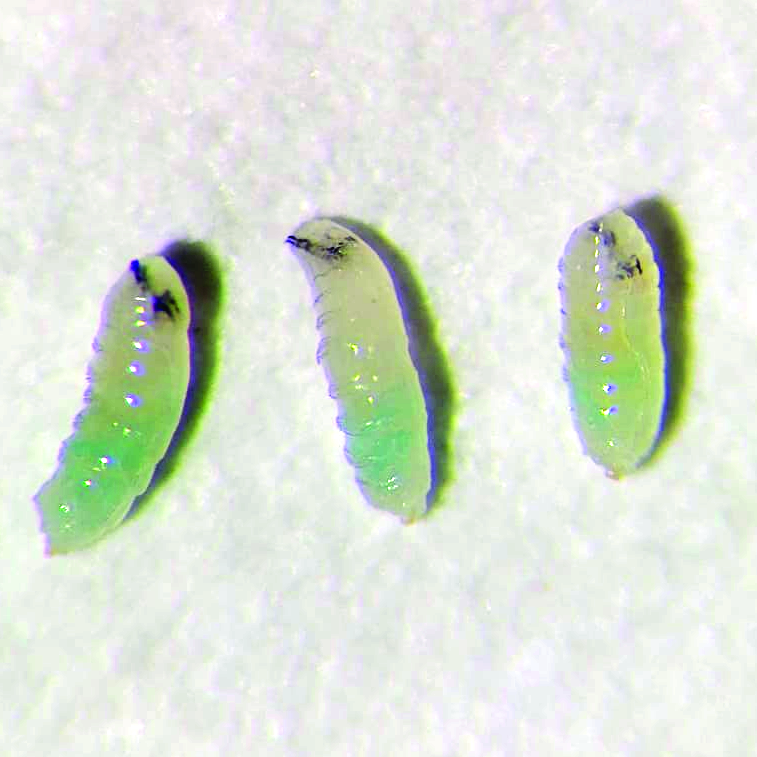

Supplement: Supplementary file 11 — Figure EV5 Source Data [file 44318_2026_775_MOESM11_ESM.zip › Figure EV5/EV5B/mCherryRNAi.tif]

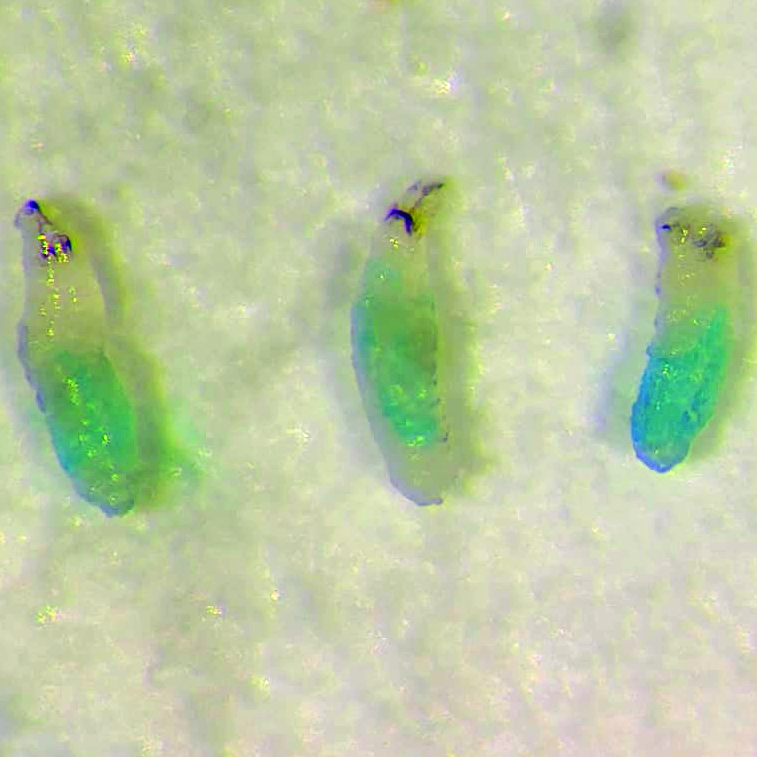

Supplement: Supplementary file 11 — Figure EV5 Source Data [file 44318_2026_775_MOESM11_ESM.zip › Figure EV5/EV5A/control.tif]

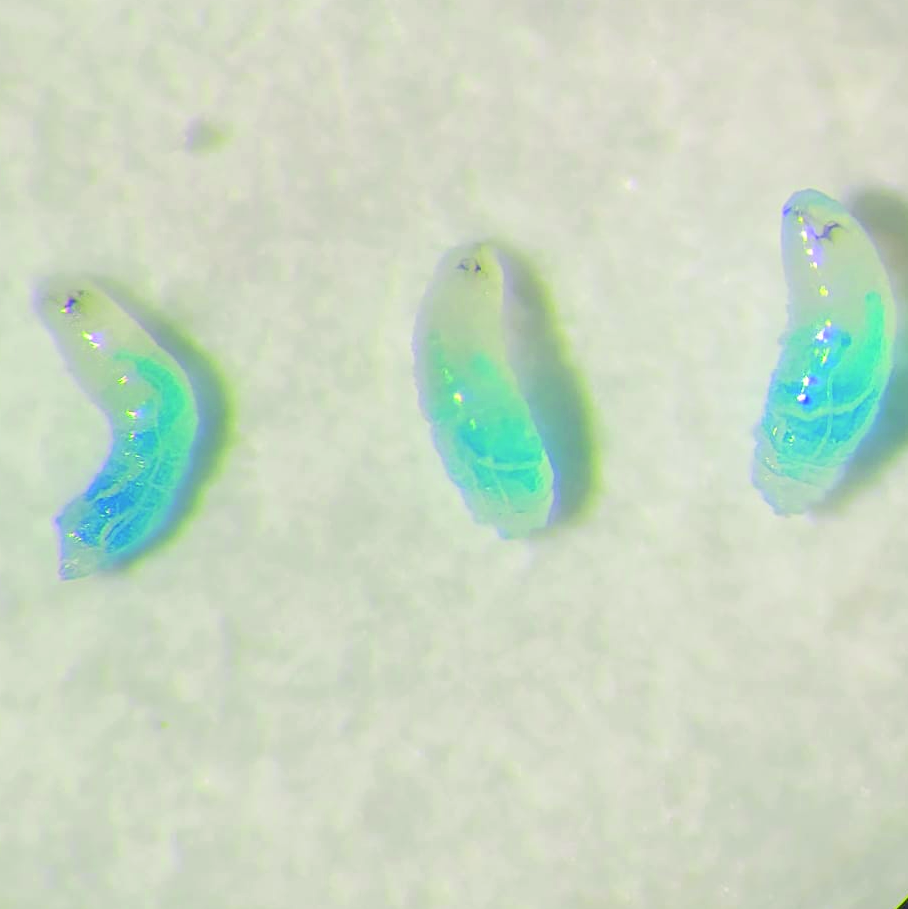

Supplement: Supplementary file 11 — Figure EV5 Source Data [file 44318_2026_775_MOESM11_ESM.zip › Figure EV5/EV5A/kir21.tif]
