# Supplementary material for: Quiescent neural stem cells transiently become neuron-like to coordinate long-range reactivation
Source: EMBO J. 2026 Apr 24;45(11):3788–807. doi: 10.1038/s44318-026-00775-3 (PMC13226747; doi:10.1038/s44318-026-00775-3)
Supplement: Supplementary file 12 — Expanded View Figures [file 44318_2026_775_MOESM12_ESM.pdf]

Expanded View Figures

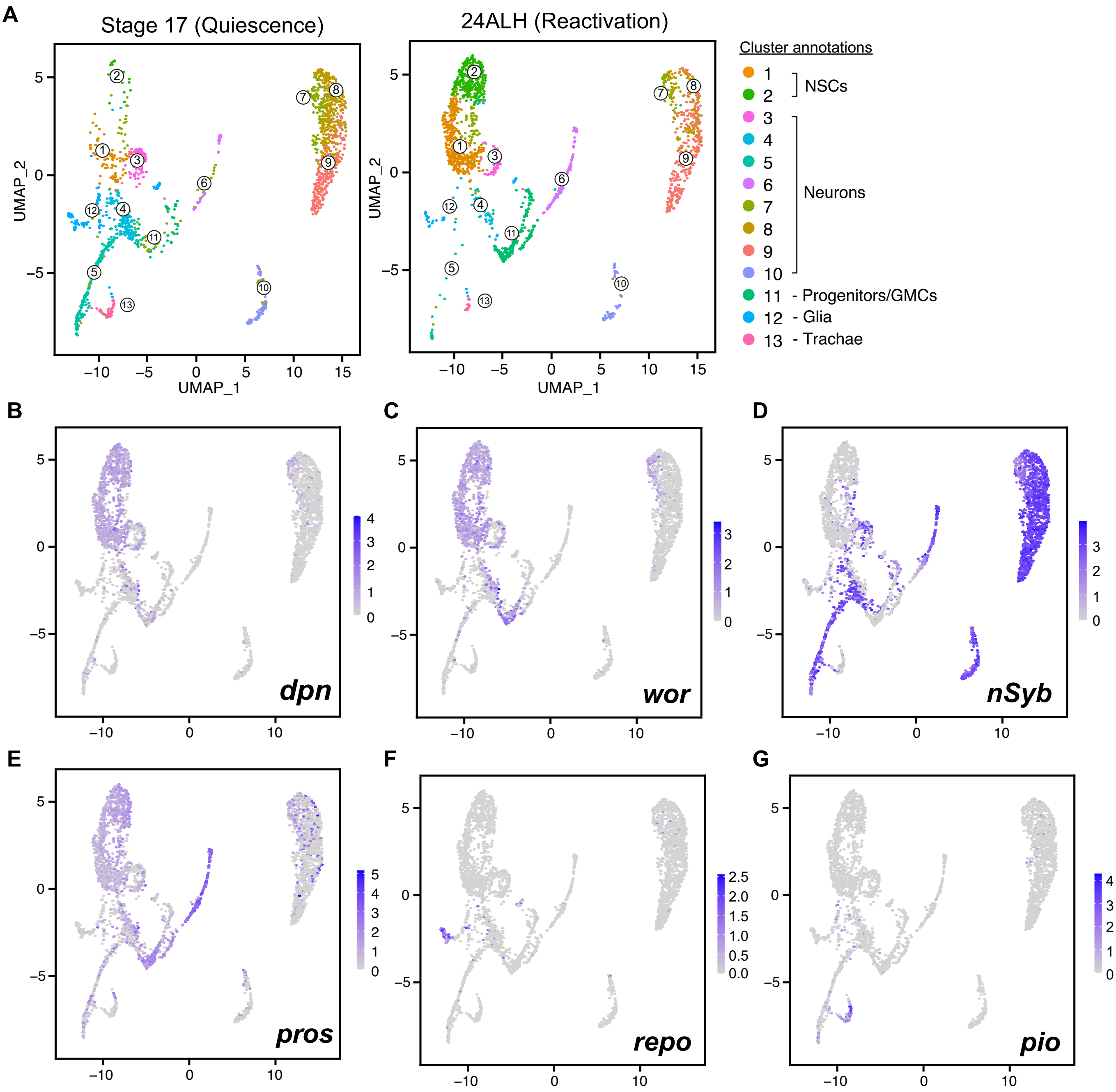

Figure EV1. Integrated scRNAseq datasets (quiescence and reactivation).

(**A**) UMAPs show different clusters assigned and distribution in each timepoint. Cells of the central nervous system (CNS) were successfully isolated and sequenced, with the majority of cells being neurons (clusters 3–10 with neuronal *Synaptobrevin* (*nSyb*) expression), as well as smaller populations of glia (cluster 12, with *reversed polarity* (*repo*) expression), NSCs (clusters 1-2, with *deadpan* (*dpn*) and *wor* expression), a population of progenitors (progenitors (cluster 11, with *prospero* expression and absence of *dpn*) and trachae (cluster 13, *grh* and *piopio* expression). (**B**) *dpn* expression, specific to NSCs, (**C**) *wor* expression, NSC-specific, (**D**) nSyb expression, specific to neurons (*nSyb* positive, *dpn* negative cells), (**E**) *pros* expression, (**F**) *repo* expression, specific to glia (**G**), *pio* expression, specific to trachea.

© The Author(s)

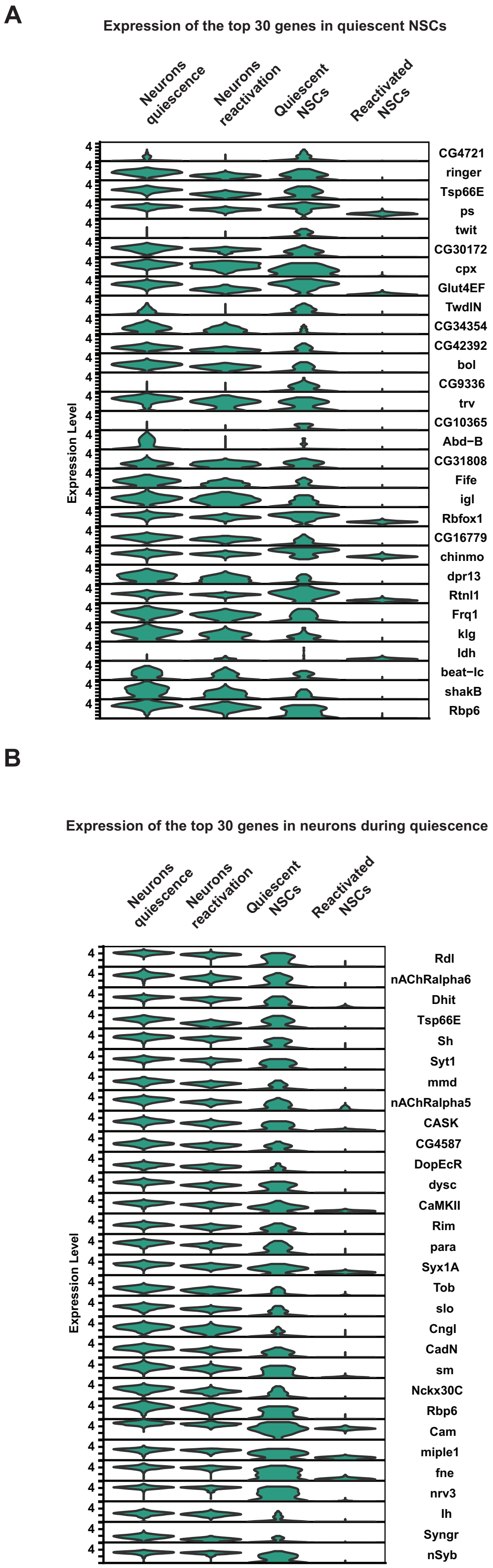
A
Expression of the top 30 genes in quiescent NSCs
Neurons quiescence
Neurons reactivation
Quiescent NSCs
Reactivated NSCs
Expression Level
CG4721
ringer
Tsp66E
ps
twit
CG30172
cpx
Glut4EF
TwdlN
CG34354
CG42392
bol
CG9336
trv
CG10365
Abd−B
CG31808
Fife
igl
Rbfox1
CG16779
chinmo
dpr13
Rtnl1
Frq1
klg
Idh
beat−Ic
shakB
Rbp6
B
Expression of the top 30 genes in neurons during quiescence
Neurons quiescence
Neurons reactivation
Quiescent NSCs
Reactivated NSCs
Expression Level
Rdl
nAChRalpha6
Dhit
Tsp66E
Sh
Syt1
mmd
nAChRalpha5
CASK
CG4587
DopEcR
dysc
CaMKII
Rim
para
Syx1A
Tob
slo
CngI
CadN
sm
Nckx30C
Rbp6
Cam
miple1
fne
nrv3
Ih
Syngr
nSyb

Figure EV2. Expression of genes in NSCs from single-cell transcriptomic data.

(**A**) Violin plots showing the average expression of the top 30 genes enriched in qNSCs. Expression levels of these 30 genes are shown in neurons (while NSCs are quiescent), qNSCs, neurons (while NSCs are reactivating), and reactivating NSCs. (**B**) Violin plots showing the average expression of the top 30 genes enriched in neurons. Expression levels of these 30 genes are shown in neurons (while NSCs are quiescent), qNSCs, neurons (while NSCs are reactivating), and reactivating NSCs.

© The Author(s)

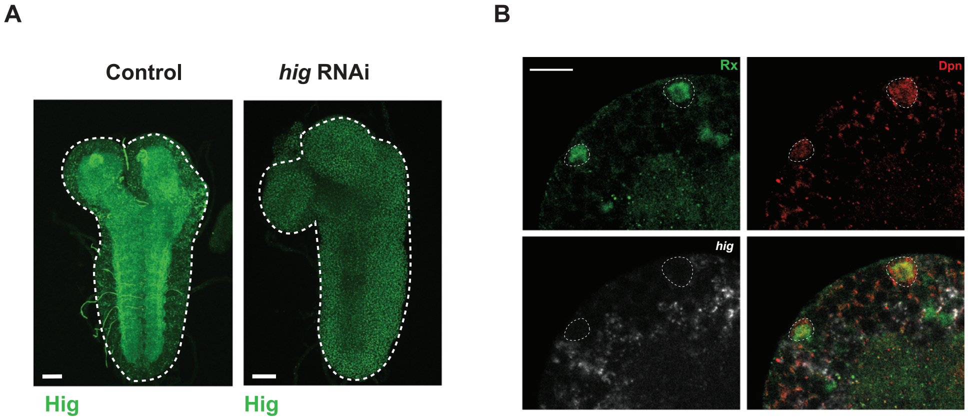

Figure EV3. Neuronal *hig* knockdown efficiency and lack of *hig* expression in mushroom body NSCs.

(**A**) Efficiency of *elav*-GAL4>higRNAi, Control: *elav*-GAL4 > w^{1118}. 0 h ALH at 29 °C, Scale bars – 20um. White dashed lines indicate the CNS outline. (**B**) *hig* is not expressed in mushroom body (MB) NSCs (Rx +) at 0 h ALH, scale bars, 10 µm, white dashed lines indicate the MB NSC. Source data are available online for this figure.

 © The Author(s)

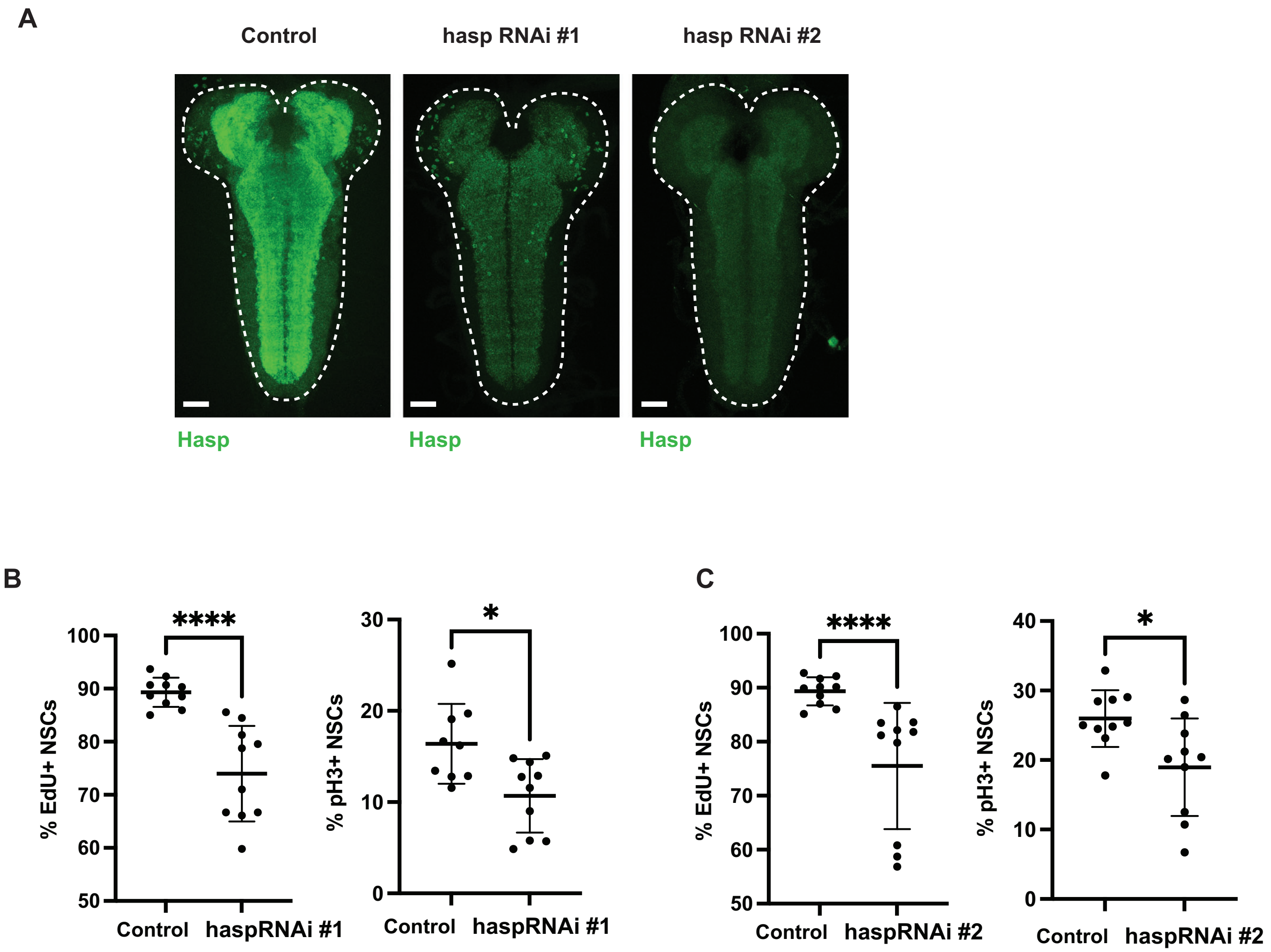

Figure EV4. Neuronal *hasp* knockdown impairs NSC reactivation.

(**A**) Efficiency of two *hasp* RNAi constructs expressed using *elav*-GAL4 with UAS-Dcr2 in the genetic background, control is *elav*-GAL4 > w^{1118}, 24 h ALH at 29 °C. Scale bars, 20 µm. White dashed lines indicate the CNS outline. (**B**, **C**) *hasp* knockdown in neurons using *elav*-GAL4 leads to impaired NSC reactivation, quantified in the tVNC at 24 h ALH, $n = 9$ control (#1,pH3), $n = 10$ control (#1 EdU, #2)/haspRNAi#1/haspRNAi#2. P value (haspRNAi #1 pH3) = 0.0182, P value (haspRNAi #2 pH3) = 0.0185. ****$P < 0.0001$, Mann–Whitney U test; error bars indicate SD, and the center is the mean. Source data are available online for this figure.

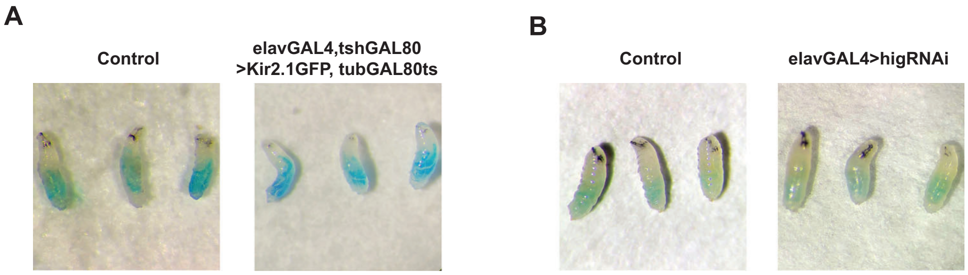

Figure EV5. Nutritional intake upon neuronal hyperpolarisation.

(**A**) Larvae continue to feed upon hyperpolarisation of brain lobe neurons after 0 h ALH (Control: *elav*-GAL4, *tsh*-GAL80 > UAS-mCD8-GFP, tubGAL80ts) and (**B**) RNAi-mediated knockdown of *hig* (Control: *elav*-GAL4 > UAS-mCherryRNAi). Images taken at 24 h ALH. Source data are available online for this figure.

 © The Author(s)
